# A causal inference framework for leveraging external controls in hybrid trials

**DOI:** 10.1093/biomtc/ujae095

**Published:** 2024-11-08

**Authors:** Michael Valancius, Herbert Pang, Jiawen Zhu, Stephen R Cole, Michele Jonsson Funk, Michael R Kosorok

**Affiliations:** Department of Biostatistics, University of North Carolina at Chapel Hill, Chapel Hill, NC 27599, United States; Product Development Data Sciences, Genentech, South San Francisco, CA 94080, United States; Product Development Data Sciences, Genentech, South San Francisco, CA 94080, United States; Department of Epidemiology, University of North Carolina at Chapel Hill, Chapel Hill, NC 27599, United States; Department of Epidemiology, University of North Carolina at Chapel Hill, Chapel Hill, NC 27599, United States; Department of Biostatistics, University of North Carolina at Chapel Hill, Chapel Hill, NC 27599, United States

**Keywords:** causal inference, double-robustness, external-control, machine-learning

## Abstract

We consider the challenges associated with causal inference in settings where data from a randomized trial are augmented with control data from an external source to improve efficiency in estimating the average treatment effect (ATE). This question is motivated by the SUNFISH trial, which investigated the effect of risdiplam on motor function in patients with spinal muscular atrophy. While the original analysis used only data generated by the trial, we explore an alternative analysis incorporating external controls from the placebo arm of a historical trial. We cast the setting into a formal causal inference framework and show how these designs are characterized by a lack of full randomization to treatment and heightened dependency on modeling. To address this, we outline sufficient causal assumptions about the exchangeability between the internal and external controls to identify the ATE and establish a connection with novel graphical criteria. Furthermore, we propose estimators, review efficiency bounds, develop an approach for efficient doubly robust estimation even when unknown nuisance models are estimated with flexible machine learning methods, suggest model diagnostics, and demonstrate finite-sample performance of the methods through a simulation study. The ideas and methods are illustrated through their application to the SUNFISH trial, where we find that external controls can increase the efficiency of treatment effect estimation.

## 1 INTRODUCTION

Establishing the causal effect of a novel intervention is imperative for making informed decisions about its adoption or approval. Randomized clinical trials (RCTs) generate robust causal evidence by ensuring the independence of treatment assignment and baseline factors. However, accessible additional data about the control treatment motivates alternative study designs that can benefit from this additional information and/or address ethical or feasibility concerns facing standard RCT enrollment, such as in rare-disease settings (Massicotte et al., 2003; Jansen-Van Der Weide et al., 2018). We consider hybrid trials where study participants are randomized to treatment via a known mechanism and externally collected control patient records (external controls) are available at analysis (Zhu et al., 2020).

**FIGURE 1:**
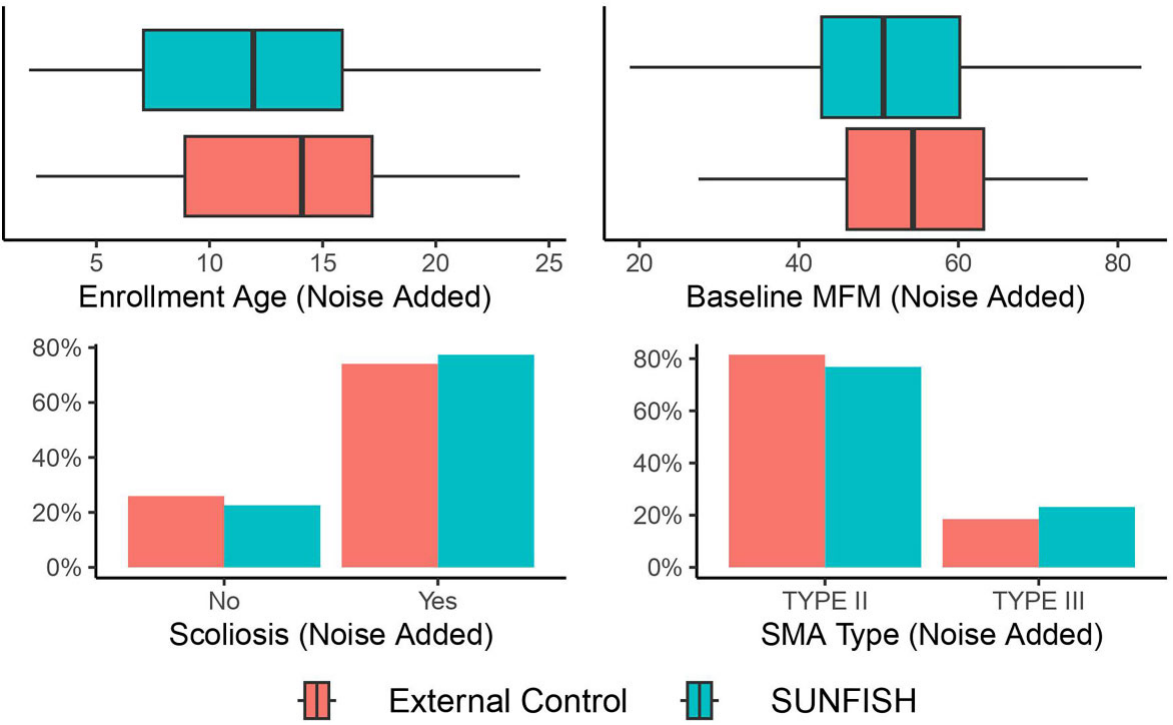
A summary of baseline characteristics believed to have an effect on a patient’s (change in) motor functioning measurement (MFM). While Scoliosis and SMA Type are similarly distributed in both samples, enrollment age and baseline MFM levels have somewhat greater degrees of discrepancy. Note: noise was added to all data points in this figure (but not in the remainder of the manuscript) to maintain first release rights for cohort-level summary in a future clinical paper; therefore, these measures should not be interpreted as the exact findings of the study and are purely for motivational purposes.

As a motivating example, we consider the analysis of SUNFISH (NCT02908685) (Mercuri et al., 2022), a two-part multi-site randomized placebo-controlled trial designed to investigate the efficacy of risdiplam on motor functioning for patients with spinal muscular atrophy (SMA), a rare disease. While the analysis was conducted using only data generated by the trial, we propose an alternative analysis incorporating external controls from the placebo arm of a Phase 2 trial of olesoxime (NCT01302600) to increase power. In Part 2 of SUNFISH, 180 patients with Type 2 and non-ambulant Type 3 SMA were randomized 2:1 to receive risdiplam or control; the primary endpoint of interest was the change in Motor Function Measure (MFM) at 12 months ($\Delta _{MFM}$). While the external controls exhibit promise as an additional source of information due to temporal and geographic similarities to the SUNFISH trial, differing distributions of important characteristics known to affect motor functioning, such as the patient’s age (Figure 1, noise added), could induce confounding. We aim to develop an analysis framework for deciding how (and if) to leverage this external control data to more precisely estimate causal effects in similar settings.

The challenges associated with using external controls in favor of a traditional RCT have been studied for at least half of a century, with Pocock’s 1976 criteria (Pocock, 1976) becoming a frequently referenced standard for evaluating the suitability of external controls. More recently, others have outlined practical considerations and potential sources of bias in hybrid trials with external controls (Zhu et al., 2020; Hall et al., 2021).

The first account of the setting through the lens of causal inference was provided by Li et al. (2023), whose foundational work outlined causal assumptions (the same as in this work) to identify the treatment effect and proposed a doubly robust estimator based on the efficient influence curve. Our work is complementary: under the same setting, we develop alternative estimators and establish theory that allows for machine learning-based estimation of the nuisance functions. Furthermore, our focus is comparatively on developing a comprehensive causal framework to guide practitioners and regulators.

Propensity score methods have been proposed to estimate the conditional probability of an individual receiving the treatment to either match external controls to study participants (Lin et al., 2018) or inversely weight external controls (Magaret et al., 2022). Others have focused on estimating the conditional average treatment effect (CATE) (Zhou and Ji, 2021). An alternative approach to incorporating external controls forgoes an explicit causal model, borrowing information from external controls through an adaptive Bayesian prior. The amount of borrowing adapts to heterogeneity between the internal and external controls, with external controls whose outcome conflicts with internal controls contributing less to the prior (Schmidli et al., 2014; Ibrahim et al., 2015; Liu et al., 2021).

Causal inference challenges in this setting are closely related to those of generalizability/transportability (Cole and Stuart, 2010; Degtiar and Rose, 2023; Shi et al., 2023). Because both settings generally necessitate and leverage some degree of comparability between multiple populations, they adopt similar assumptions and proposed estimators (Dahabreh et al., 2019a; Shi et al., 2023). However, the analysis goals typically differ (efficiency versus target population coverage), and differing target parameters and data produce distinct estimators.

In this work, we develop a causal framework intended to assist practitioners in conducting the analysis of a randomized trial incorporating external controls, drawing focus to two primary themes. (**1**) Randomization to treatment is no longer (fully) under investigator control. (**2**) Adjustment for imbalances due to a lack of randomization can increase dependence on statistical models. To address (**1**), we present a causal inference framework for rigorously defining the target parameter, assessing assumptions via graphical criteria, choosing between classes of estimators, and checking model assumptions. Leveraging advances in statistical causal inference theory (Hines et al., 2022), we alleviate (**2**) by building upon the previously proposed doubly robust estimator (Li et al., 2023) and proving conditions under which the estimator is efficient and asymptotically normal even when machine learning methods are used for the nuisance functions, an important advance that allows for valid inference under broader data-generating mechanisms. The variance reduction and double-robustness are explored through a simulation study, and the entire causal framework is demonstrated through a study of patients with spinal muscular atrophy.

## 2 SETTING

In a hybrid trial design with external controls, data from two sources are used to estimate a treatment effect. Precisely defining this objective requires carefully answering two questions. (**1**) *What two populations generated the data?* Differences between these populations govern both the capacity to combine the two data sources and the methods to do so accurately and efficiently. (**2**) *What definition of treatment effect?* Heterogeneity of individual treatment effects causes average treatment effects to vary depending on the population composition.

Denote the outcome of interest by *Y*, baseline covariates collected both in the trial and for external controls by *Z*, and a binary treatment by *A* ($a=1$ is the experimental treatment and $a=0$ is the control treatment, possibly a placebo, active control, or other intervention). For ease of exposition, we will refer to the dichotomous treatments as treatment and control, respectively. We use *D* to indicate the source of the data ($d = 1$ for RCT and $d =0$ for external control). Definitions of populations relevant to inference vary across contexts and disciplines; we adopt definitions commonly used within the causal inference literature (Degtiar and Rose, 2023). The *study sample* consists of $n_{rct}$ iid copies of the random vector $(Y, Z, A, D=1)$ sampled from the (possibly hypothetical) population from which the study sample is a simple random sample. Investigators also have access $n_{ec}$ iid copies of the random vector $(Y, Z, A, D=0)$ from a second sample of individuals, the external controls. These external controls might have come from one of several sources, including previous clinical trials (Darras et al., 2021) and electronic health record (EHR) systems (Carrigan et al., 2020), and it is implicitly understood that they have been selected to satisfy the inclusion/exclusion criteria of the trial and were administered the same control treatment as the internal controls. These individuals are viewed as a simple random sample from another population, titled the *external control population*. Presently, no assumptions are made about the relationship between the external control and study populations. For $a \in \lbrace 0, 1\rbrace$, we posit the existence of potential outcomes $Y^a$ that represent the outcome that would have been observed under the intervention to receive treatment $A = a$ (Rubin, 2005). We make three assumptions expected to hold in an RCT (**A1**-**A3**) and a fourth (**A4**) motivated by the discussion in Section 3.

(**A1**) *Consistency*: $Y^a = Y$ if $A = a$ for $a \in \lbrace 0,1\rbrace$.

(**A2**) *Exchangeability*: $Y^a \perp A | D = 1$ for $a \in \lbrace 0, 1\rbrace$.

(**A3**) *Positivity of treatment assignment*: $0 < \mathrm{Pr}(A = 1 | D = 1) < 1$.

(**A4**) *Mean-exchangeability across populations*: there exists $X \subset Z$ such that $E[Y^0 | D = 1, X=x] = E[Y^0 | D = 0, X=x]$ for all *x* with $p(x|D = 1) > 0$.

### 2.1 Target parameter

While a number of different parameters might be of scientific interest, in this paper we focus on the ATE in the study population, a natural choice because it is typical parameter that would have been estimated in an RCT without external controls. This treatment effect is defined as the expected difference between outcomes if the study population were assigned the treatment versus if the study population were assigned the control: $\tau \equiv E[Y^1 - Y^0 | D = 1]$. When treatment effects are heterogeneous and the external control population differs from the study population, $\tau$ may differ from $E[Y^1 - Y^0 | D = 0]$ or $E[Y^1 - Y^0]$. Identification of these two parameters also requires assumptions beyond those for $\tau$. Because $D = 0$ implies $A = 0$ and study participants are randomized to treatment, we can equivalently define $\tau$ as $E[Y^1 - Y^0 | A = 1]$, which is the average treatment effect among the treated (ATT).

## 3 CAUSAL IDENTIFICATION

Under **A1**-**A3**, $\tau = E[Y | A = 1, D = 1] - E[Y | A = 0, D = 1]$ so that the difference of mean outcomes between the treatment and internal control arms is a consistent estimator. Such identification is possible because, within the RCT ($D = 1$), patients are randomized to treatment. However, this identification (linking of counterfactual parameter with the observable data distribution) does not incorporate external controls, and thus no efficiency has been gained. Incorporating of external controls comes at the expense of randomization: while $\mathrm{Pr}(A = 1 | D = 1)$ is under investigator control, $\mathrm{Pr}(A = 1) = \mathrm{Pr}(A = 1 | D = 1) \mathrm{Pr}(D = 1)$, and *D* is not randomly assigned. Consequently, there is no justification, without further assumptions, for $Y^0 | D=1$ and $Y^0 | D = 0$ being equal in distribution (or even moments), implying that internal and external control outcomes may differ systematically.

To address this, we show how knowledge of the underlying causal structure generating the data, as formalized through a causal graph, can identify a sufficient set of variables $X \subset Z$ that, when conditioned on, recover exchangeability of $Y^0$ across the study and external control populations. As a simple example, consider a case where an outcome *Y* is a causal descendent of a covariate *X* plus an independent noise term so that $Y = f(X, \epsilon )$. If, across populations, *f* and the distribution of $\epsilon$ were fixed but the distribution of *X* shifted, then the distribution and expectation of *Y* (generally) differ across the populations. Relating this to hybrid trials, discrepancies in the distribution of causes of the outcome between the study and external control populations are responsible for differing distributions of $Y^0 | D = 1$ and $Y^0 | D = 0$.

Graphical conditions for identifying average treatment effects in the cases of generalizability (Dahabreh et al., 2019b) and transportability (Bareinboim and Pearl, 2013) have been developed. However, causal graphs for this external control setting pose unique challenges. First, whereas a causal graph is typically defined with respect to a specific population, in the external control setting data is sampled from two, possibly distinct, populations. Furthermore, while the typical goal of causal graphs is to verify if $\lbrace Y^0, Y^1\rbrace \perp \!\!\! \perp A | X$, in the external control setting the objective is instead to verify if $Y^0 \perp \!\!\! \perp D | X$ for some $X \subset Z$.

Motivated by these unique characteristics, we propose a variant of SWIGs (Richardson and Robins, 2013) inspired by selection diagrams (Bareinboim and Pearl, 2013), which are modified causal graphs that determine the transportability of causal relations. Let $\mathbf {G}$ denote a causal graph (SWIG), which encodes the investigator’s knowledge of the causal process and contains all variables *Z* (possibly unmeasured) that are direct causes of *Y* (i.e. have arrows pointing from *Z* to *Y*). A *selection SWIG*  $\mathbf {D}$ is created as follows:

- Every edge in $\mathbf {G}$ is also an edge in $\mathbf {D}$;
-
$\mathbf {D}$ contains an extra edge, $S_{Z_i} \longrightarrow Z_i$, whenever $p(z_i | v, d = 1) \ne p(z_i | v, D = 0)$ for some collection of variables $V \subset Z \backslash Z_i$; and
-
$\mathbf {D}$ contains an extra edge, $S_{Y} \longrightarrow Y$, whenever $p(y | z, d = 1) \ne p(y | z, d = 0)$.

Intuitively, this process adds *S* variables to pinpoint discrepancies in the study and external control distributions, which are informative for understanding if $Y^0 | D = 1 \ne Y^0 | D = 0$. The following result, where conditional independence statements are evaluated on $\mathbf {G}$ using the rules of *d*-separation, formalizes this by demonstrating how this process identifies which variables are sufficient so that, after their adjustment, the distribution of $Y^0$ is balanced.

Theorem 1 *Let the selection SWIG*  $\mathbf {D}$  *be constructed as above and let $X \subset Z$. Then:*
 

- *If*  $Y^0 \perp S | X$, *then*  $p(Y^0 | X, D = 1) = p(Y^0 | X, D = 1)$.
- *If*  $Y^0 \not\perp S | X$, *then there exists some two distributions compatible with*  $\mathbf {G}$  *such that*  $p(Y^0 | X, D = 1) \ne p(Y^0 | X, D = 1)$*, even if*  $p(y^0 | z, D = 1) = p(y^0 | z, D = 0)$.

To identify $E[Y(0) | D = 1]$ using both internal and external controls, we leverage the preceding discussion to make an assumption about the comparability of internal and external controls (**A4**). Assumption 4 states that while $Y^0$ might systematically differ between the study and external control population, once differences in the distribution of *X* are accounted for, the expectation of $Y^0$ is the same across populations. While investigators do not randomize individuals to *D* (and thus treatment *A*), once *X* is conditioned upon, the mean of $Y^0$ is independent of *D*, mimicking randomization. By Theorem 1, **A4** is satisfied if there exists some set of variables $X \subset Z$ such that $Y^0 \perp \!\!\! \perp S | X$ on the selection SWIG $\mathbf {D}$.

Equipped with these causal assumptions, the target parameter $\tau$ can be identified with the observed data distribution. By conditioning, $\tau$ can be written as $E[Y^1 | D = 1] - E_{X | D = 1}[E[Y^0 | D = 1, X]]$. The first term, as noted earlier, is equivalent to $E[Y | A = 1]$ under **A1**-**A3**. The second term can be shown, using **A1**-**A4**, to be equal to $E_{X | D = 1}[E[Y | A = 0, X]]$.

Therefore, we have that


(1) \begin{eqnarray*}
\tau = E[Y | A = 1] - E_{X | D = 1}[E(Y | A = 0, X)].
\end{eqnarray*}

## 4 ESTIMATION

In what follows, define $m_a(x)$ as $E[Y | A=a, X = x]$, $\pi _a(x)$ as $\mathrm{Pr}(A = 1 | X = x, D = 1)$ (*the treatment propensity score*), and $\pi _d(x)$ as $\mathrm{Pr}(D = 1 | X = x)$ (*the study propensity score*). Corresponding estimators are denoted as $\widehat{m}_a(x)$, $\widehat{\pi }_a(x)$, and $\widehat{\pi }_d(x)$, respectively. From Equation 1, $\tau$ is identified as the difference between $\mu_1 := E[Y | A = 1]$ and $\mu _0 := E_{X | D = 1}[E[Y | A=0, X]]$. When *X* is high dimensional or contains continuous covariates, simple nonparametric estimators of $\mu _0$ are no longer feasible, and thus models are needed for the “nuisance” functions $m_0(x)$ and $\pi _d(x)$. Thus, distributional differences between internal and external controls, from a statistical perspective, heighten model dependency. In this section, we outline three primary classes of estimators that will be applied to the SMA example.

### 4.1 Outcome based models

The quantity $\mu _0$ averages the expected outcome of controls given baseline covariates *X* over the distribution of *X* in the study. Because it depends on the data only through $m_0(x)$ and the marginal distribution of *X* in the study population, we can consider a simple plug-in estimator, where a regression model $\widehat{m}_0(x)$ is fit on the internal and external control data and its predictions are averaged over the empirical distribution of *X* from the study sample:


\begin{eqnarray*}
\widehat{\mu }_{0,om} = n_{rct}^{-1} \sum _{i=1}^n D_i \widehat{m}_0(X_i).
\end{eqnarray*}

In the causal inference literature, this estimator is frequently referred to as standardization or *g*-computation (Hernan and Robins, 2020). For this estimator, the benefit of incorporating external controls comes from a more refined estimate of $m_0(x)$.

### 4.2 Study propensity score-based models (IPDW)

As an alternative to modeling the outcome, $\pi _d(x)$ can be used to re-weight the outcomes of the controls so that their distribution of *X* in this re-weighted population is the same as the treated. This idea is analogous to inverse propensity score weighting in causal inference. The usual propensity score, $\pi (x) := \mathrm{Pr}(A = 1 | x)$ is equal to $\pi _a(x) \pi _d(x)$. While $\pi _a(x)$ is known by trial-design, $\pi _d(x)$ is unknown, and so the treatment assignment mechanism across the entire sample is unknown. After estimating $\widehat{\pi }_d(x)$ and $\widehat{\pi }_a(x)$, we consider the estimator $\widehat{\mu }_{0, ipdw} = n_{rct}^{-1} \sum _{i=1}^n \widehat{W}^{ipdw}_i Y_i$, where


\begin{eqnarray*}
\widehat{W}^{ipdw}_i = \frac{(1-A_i) \widehat{\pi }_d(X_i)}{[1 - \widehat{\pi }_a(X_i)] \widehat{\pi }_d(X_i) + [1 - \widehat{\pi }_d(X_i)]} .
\end{eqnarray*}

Methods that handle extreme weights in $\widehat{\mu }_{0, ipdw}$ or leverage $\widehat{\pi }_d$ for matching instead of weighting are discussed in Section 8 of the Supporting Information.

### 4.3 Efficient estimators

The proposed estimators in the previous sections, which are plug-in estimators for $\mu _0$ based on Equation 1 and its inverse propensity weighted analog, incorporate a model for either $m_0(x)$ or $\pi _d(x)$. Heuristically, more precise estimators for $m_0(x)$ or $\pi _d(x)$ can produce a less variable estimator of $\tau$. Since the goal of incorporating external controls is to increase efficiency in estimating $\tau$, a relevant aim is to construct estimators that achieve the greatest reduction in variance, for which we turn to semiparametric efficiency theory.

Any regular and asymptotically linear (RAL) estimator for $\tau$ has an asymptotic variance of at least $\mathrm{B}_{\tau } \equiv E[\phi (O; \mathbb {P})^2]$, where $O = (Y, X, A, D)$ and $\phi (O; \mathbb {P})$ is the *efficient influence curve* (EIC) for $\tau$ (Kosorok, 2008). The EIC in this setting is (Li et al., 2023)


(2) \begin{eqnarray*}
\phi (O; \mathbb {P}) &=& \frac{1}{q} \bigg \lbrace D [m_1(X) - m_0(X) - \tau ] \\
&& + \frac{DA}{\pi _a(X)}[Y - m_1(X)] - W^{dr} [Y - m_0(X)] \bigg \rbrace , \\
\end{eqnarray*}

where $q \equiv \int \pi _d(x) d\mathbb {P}$, $r(X) \equiv \mathrm{Var}(Y^0 | X, D = 1)/\mathrm{Var}(Y^0 | X, D = 1)$ and where


\begin{eqnarray*}
W^{dr} \equiv \frac{D(1-A) \pi _d(X) + (1-D) \pi _d(X) r(X)}{\pi _d(X)[1-\pi _a(X)] + [1-\pi _d(X)]r(X)}.
\end{eqnarray*}

We consider two types of estimators based upon the form of $\phi (O; P)$, where we write $\phi$ as a function of a distribution *P* to highlight that it depends on the nuisance functions $m_1(x)$, $m_0(x)$, $\pi _d(x)$, and $r(x)$ as well as the target parameter $\tau$. If these functions were known, then (after uncentering) the estimator $n^{-1} \sum _{i=1}^n \phi (O_i, \mathbb {P})$ would achieve the efficiency bound. Therefore, the first influence-curve-based estimator, as proposed by Li et al. (2023) and based upon an estimating equation, plugs in fitted models for the nuisance functions:


\begin{eqnarray*}
\widehat{\tau }_{aipw} &=& \frac{1}{n}\! \sum _{i=1}^n \frac{1}{\widehat{q}} \bigg \lbrace \! D [\widehat{m}_1(X) - \widehat{m}_0(X)] + \frac{DA}{\widehat{\pi }_a(X)}[Y \! - \widehat{m}_1(X)] \\
&&- \widehat{W}(A, D, X) [Y - \widehat{m}_0(X)] \bigg \rbrace ,
\end{eqnarray*}

where $\widehat{q} = \frac{n_{rct}}{n}$ and


\begin{eqnarray*}
\widehat{W}^{dr} = \frac{D(1-A) \widehat{\pi }_d(X) + (1-D) \widehat{\pi }_d(X) \widehat{r}(X)}{\widehat{\pi }_d(X)[1-\widehat{\pi }_a(X)] + [1-\widehat{\pi }_d(X)]\widehat{r}(X)}.
\end{eqnarray*}

An alternative strategy is through targeted maximum likelihood estimation (TMLE) (Van der Laan and Rose, 2011). While the two approaches here produce asymptotically equivalent estimators, TMLE is a plug-in estimator and thus respects the boundaries of the parameter space for all sample sizes, potentially leading to improved performance in small sample sizes. Instead of using fitted models $\widehat{m}_1(x)$ and $\widehat{m}_0(x)$ to form a simple plug-in estimator of $\tau$ (as with $\widehat{\mu }_{0, om}$), the models are fluctuated so that $n^{-1} \sum _{i=1}^n \phi (O_i, \widehat{\mathbb {P}}^{*}) = 0$, implying that it, like $\widehat{\tau }_{aipw}$, solves the efficient influence curve estimating equation. This is accomplished through fitting a logistic regression model of the outcome (scaled to [0,1]) on $h(D, A, X)$ with an offset given by the logit of $\widehat{m}_A(X)$, where


\begin{eqnarray*}
h(D, A, X) &\equiv & \frac{1}{q} \bigg \lbrace \frac{DA}{\widehat{\pi }_a(X)} \\
&&- \frac{D(1-A) \widehat{\pi }_d(X) + (1-D)(1-A) \widehat{\pi }_d(X) \widehat{r}(X) }{\widehat{\pi }_d(X) [1 - \widehat{\pi }_a(X)] + [1- \widehat{\pi }_d(X)] \widehat{r}(X)} \bigg \rbrace .
\end{eqnarray*}

For each observation, this model produces predictions under the settings $A = 1$ and $A = 0$, which defines our updated models $\widehat{m}_1^{*}(x)$ and $\widehat{m}_0^{*}(x)$. Because convergence occurs after only one update iteration, the TMLE estimator, $\widehat{\tau }_{tmle}$, is just a plug-in estimator using the updated models $\widehat{m}_1^{*}(x)$ and $\widehat{m}_0^{*}(x)$: $\widehat{\tau }_{tmle} = n_{rct}^{-1} \sum _{i=1}^n D_i \big [ \widehat{m}_1^{*}(X_i) - \widehat{m}_0^{*}(X_i) \big ]$. Further details are presented in Section 9 of the Supporting Information.

### 4.4 Inference

For the wide adoption of a method and its regulatory approval, inferential properties should be established, including conditions under which the estimator is consistent and how measures of uncertainty can be constructed. The consistency of the first two approaches is contingent upon their nuisance functions estimates: when $\widehat{m}_0$ is consistent for $m_0$, $\widehat{\mu }_{0,om}$ is consistent for $\mu _0$, and when $\widehat{\pi }_d(x)$ is consistent for $\pi _d(x)$, $\widehat{\mu }_{0, ipdw}$ is consistent for $\mu _0$. While this seemingly favors flexible, data-adaptive methods to avoid misspecifying $m_0(x)$ and $\pi _d(x)$, doing so generally leads to slow rates of convergence and a poorly understood limiting distribution for $\widehat{\tau }$. Therefore, parametric models and confidence intervals constructed via the nonparametric bootstrap are recommended.

The trade-off between reducing bias through using flexible models and maintaining valid $\sqrt{n}$ inference motivates the usage of $\widehat{\tau }_{aipw}$ and $\widehat{\tau }_{tmle}$, which possess several desirable statistical properties. First, the models are doubly robust in the sense that $\widehat{\tau }$ converges in probability to $\tau$ if either $\widehat{m}_0(x)$ converges in probability to $m_0(x)$ and $\widehat{m}_1(x)$ converges in probability to $m_1(x)$ or if $\widehat{\pi }_d(x)$ converges in probability to $\pi _d(x)$ (Li et al., 2023). Furthermore, when cross-fitting is used, $\widehat{\tau }_{aipw}$ and $\widehat{\tau }_{tmle}$ are both efficient and asymptotically normally distributed, even when the nuisance functions are estimated at slower rates, allowing for more flexible estimation of these functions using popular machine learning methods.

Theorem 2 *Under certain regularity conditions (Supporting Information, Section 10)*, $\widehat{\tau }_{dr}$, *meaning either*  $\widehat{\tau }_{aipw}$ or $\widehat{\tau }_{tmle}$, *satisfies*  $\sqrt{n} (\widehat{\tau }_{dr} - \tau ) \longrightarrow N(0, E[\phi (O, \mathbb {P})])$. *Therefore, $\widehat{\tau }_{dr}$ is root-n consistent, semiparametric efficient, and asymptotically normal with asymptotically valid confidence intervals given by*  $\widehat{\tau }_{dr} \pm 1.96 \sqrt{\widehat{\mathrm{var}}(\phi (O, \widehat{\mathbb {P}})) / n}$.

## 5 MODEL AND ASSUMPTION ASSESSMENTS

The analysis of causal effects in hybrid trials with external controls substantively differs from that of an RCT because additional assumptions and techniques are needed to identify and estimate the average treatment effect. Consequently, as we discuss now and demonstrate in our SMA example in Section 7, model and assumption assessments can play a critical role in validating the trustworthiness of results.

### 5.1 Covariate imbalance

Covariate imbalance, here the dissimilarity in covariate distributions of the control arms, plays a critical role in the identification and estimation of $\tau$. As shown in Section 3, covariate imbalances in the causes of the outcome are responsible for $Y(0)$ differing in distribution across control arms. This in turn necessitates covariate adjustment to identify $\tau$ as a function of the external control distribution. Covariate imbalance also has important implications in variance reduction: the efficiency gain attributable to external controls is proportional to $E[(1-\pi _d(X))|D=1]$, so that variance gains are largest when there is complete covariance balance. Furthermore, covariate imbalance increases model dependency because inferences are extrapolated to regions with limited common support. Conversely, under complete covariate balance, the methods discussed in Section 4 are typically still consistent for $\tau$ even when the functional form of $m_0$ is incorrectly specified, permitting a degree of model insensitivity.

Due to these factors, numerical assessment is recommended to explicate the degree of covariate balance. One measure of covariate balance is the normalized difference, $\widehat{\Delta }_{x} = \sqrt{2 (\overline{X_1} - \overline{X}_0)^T(\widehat{\Sigma }_1 + \widehat{\Sigma }_0)^{-1} (\overline{X_1} - \overline{X}_0)}$, where $\overline{X}_d$ and $\widehat{\Sigma }_d$ are the sample mean and covariance matrix of *X* in control arm *d*. Alternatively, imbalance can be judged using the propensity score by calculating $\widehat{\Delta }_{\pi _d} = n_{rct}^{-1} \sum _{i=1}^n D_i \widehat{\pi }_d(X_i) - n_{ec}^{-1} \sum _{i=1}^n (1-D_i) \widehat{\pi }_d(X_i)$. When the distribution of *X* is the same in the study and external control populations, we expect both $\widehat{\Delta }_x$ and $\widehat{\Delta }_{\pi _d}$ to be 0. Larger values are consistent with less covariate balance, and $\widehat{\Delta }_{\pi _d} > 0.25$ has previously been suggested as a threshold in the context of generalizability (Stuart et al., 2011). Hypothesis tests of these statistics are not recommended as departures from 0 do not invalidate the methods of Section 4. However, reporting these (or related) summaries is useful because it provides a difficulty measure for the statistical task of adjusting for imbalance.

### 5.2 Assumption 4

The Selection SWIG of Section 3 provides a theoretical justification for **A4** based upon background knowledge of the causal process. While assumptions within causal inference are often untestable, **A4**, when combined with the consistency assumption, implies that $u(X) := E[Y | A = 0, D = 1, X] - E[Y | A = 0, D = 0, X] = 0$, a so-called “testable implication” that can also appear in transportability settings (Degtiar and Rose, 2023). This is an assumption that two (nonparametric) regression functions are equal. While some statistical tests are applicable (Racine et al., 2006; Luedtke et al., 2019), decisions about whether to incorporate external controls on the basis of such tests are inadvisable due to their limited power in sample sizes common to these trials and challenges posed by post-selection inference. Importantly, a failure to reject **A4** through these tests should not be taken as proof in its favor. Alternatively, we recommend visual diagnostics as supporting information following the incorporation of external controls. Because $E[Y | A = 0, D, \pi _d(X)] = E[Y | A = 0, \pi _d(X)]$ under **A4**, one useful one-dimensional diagnostic is plotting the mean outcomes of internal controls against external controls with similar estimated study propensity scores.

Instead of scrutinizing **A4** in isolation, sensitivity analyses can directly examine how its violation incurs bias in estimating $\tau$. When the nuisance functions are estimated consistently, the asymptotic bias of the estimators is $E[\text{Pr}(D=0|A=0,X)u(X) | D= 1]$. The first term highlights a fundamental trade-off: covariate balance and more external controls improve efficiency but heighten sensitivity to bias. When the terms are independent [non-adversarial $u(X)$], the bias is upper-bounded by $\text{Pr}(D=0|A=0) E[u(X)|D=1]$, which can be estimated using either a pre-determined model (possibly a constant *B*) or an estimator (fitting models for $E[Y | A = 0, D = d, X]$) for $u(x)$. One criteria for evaluating robustness is to determine the minimum magnitude *B* required to shift the confidence interval for $\tau$ so that the treatment effect is no longer deemed significant. In Section 11 of the Supporting Information, we also provide an upper bound on $E[u(X)|D=1]$ that can be useful in settings where the residual variation in the outcome is small relative to the treatment effect.

## 6 SIMULATION STUDY

We perform a simulation study to evaluate the properties of the proposed estimators, focusing on when the distribution of *X* differs between internal and external controls. We evaluate the estimators in terms of their bias and variance, whether their confidence intervals achieve their stated coverage (Supporting Information, Section 13), and whether they achieve higher power while controlling Type-1 error.

In concordance with our SMA application, we simulate a trial investigating a continuous endpoint with 150 RCT patients randomized 2:1 to treatment and 50 external controls. All simulations are run for 1000 replicates. Full details of the data-generating mechanism and nuisance function models are available in the Supporting Information (Section 12); importantly, $\pi _d(x)$ is non-constant so that $A \not\perp D$. To highlight how certain settings benefit from the usage of flexible estimators for the nuisance functions, we evaluate the aforementioned properties when the nuisance functions have a known functional form, are incorrectly specified, or are adaptively estimated using machine learning methods. In Setting 1, all nuisance functions are estimated with correctly specified linear models. In Setting 2, an incorrectly specified linear model is used for $\pi _d(x)$ while Random Forests are used for $m_a(x)$. In Setting 3, incorrectly specified linear models are used for $m_a(x)$ while a Random Forest is used for $\pi _d(x)$. In Setting 4, incorrectly specified linear models are used for both $m_a(x)$ and $\pi _d(x)$. Both $m_a(x)$ and $\pi _d(x)$ are modeled with Random Forests in Setting 5.

To estimate $\tau$, we compare an RCT-only covariate-adjusted AIPW estimator ($\widehat{\tau }_{rct}$) with the four proposed estimators ($\widehat{\tau }_{om} \equiv \widehat{\mu }_1 - \widehat{\mu }_{0, om}$, $\widehat{\tau }_{ipdw} \equiv \widehat{\mu }_1 - \widehat{\mu }_{0, ipdw}$, $\widehat{\tau }_{aipw}$, and $\widehat{\tau }_{tmle}$). Both doubly robust approaches correspond to their cross-fit (10-fold) variants. For $\widehat{\tau }_{om}$ and $\widehat{\tau }_{ipdw}$, 95% confidence intervals were constructed via the non-parametric bootstrap. Confidence intervals were generated for $\widehat{\tau }_{aipw}$ and $\widehat{\tau }_{tmle}$ using the closed-form formula from Theorem 1.

**Bias and variance**: With correctly specified parametric models (Setting 1), all external control approaches are unbiased and exhibit less variability (Table 1). However, performance gains are not guaranteed under departures from correctly specified parametric models. In Settings 2 and 3, where one nuisance function is incorrectly specified, the singly robust approaches ($\widehat{\tau }_{om}$ and $\widehat{\tau }_{ipdw}$) are biased. Conversely, the doubly robust approaches (with one nuisance function estimated flexibly and the other missecified) have minimal bias but lower MSEs than $\widehat{\tau }_{rct}$. Furthermore, while double robustness offers no protection against bias when both nuisance functions are incorrectly specified (Setting 4), the data-adaptive estimation of both functions (Setting 5) leads to estimates with minimal bias and variability, highlighting the inferential gains that are possible through flexibly modeling the nuisance functions.

**TABLE 1:** Results (bias, mean-squared error, and 95% confidence interval coverage) of the simulation.

	$\widehat{\tau }_{rct}$			$\widehat{\tau }_{om}$			$\widehat{\tau }_{ipdw}$			$\widehat{\tau }_{aipw}$			$\widehat{\tau }_{tmle}$		
	Bias	MSE	Cov.	Bias	MSE	Cov.	Bias	MSE	Cov.	Bias	MSE	Cov.	Bias	MSE	Cov.
Setting 1	$-$ 1.8e-03	0.31	0.96	2.5e-04	**0.22**	0.93	$-$ 2.9e-03	0.24	0.93	$-$ 4.2e-04	0.23	0.95	**6.6e-06**	**0.22**	0.96
Setting 2	**3.4e-02**	0.31	0.95	−	−	−	4.0e-01	0.38	0.86	2.0e-01	**0.25**	0.94	2.2e-01	**0.25**	0.94
Setting 3	**3.6e-02**	0.34	0.95	4.1e-01	0.38	0.85	−	−	−	1.1e-01	**0.24**	0.96	6.8e-02	**0.24**	0.96
Setting 4	**2.5e-02**	**0.34**	0.96	4.0e-01	0.39	0.85	4.0e-01	0.39	0.86	4.2e-01	0.42	0.86	4.1e-01	0.40	0.88
Setting 5	**−4.7e-03**	0.32	0.95	2.2e-01	0.24	0.93	4.8e-02	0.22	0.94	5.8e-02	**0.21**	0.96	6.3e-02	**0.21**	0.96

In Setting 1, all nuisance functions are estimated with correctly specified linear models. In Setting 2, an incorrectly specified linear model is used for $m_a(x)$. In Setting 3, incorrectly specified linear models are used for $\pi _d(x)$ while Random Forests are used for $m_a(x)$. In Setting 3, incorrectly specified linear models are used for $m_a(x)$ while a Random Forest is used for $\pi _d(x)$. In Setting 4, incorrectly specified linear models are used for both $m_a(x)$ and $\pi _d(x)$. Both $m_a(x)$ and $\pi _d(x)$ are modeled with Random Forests in Setting 5.

**FIGURE 2:**
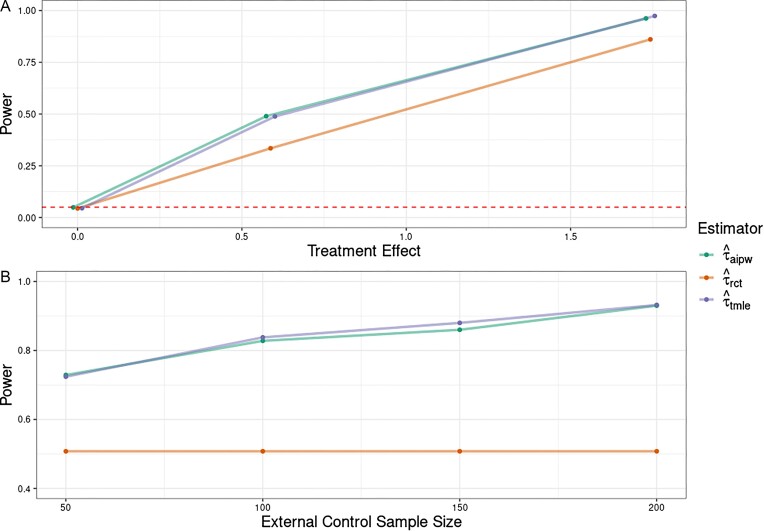
(**A**) Power across different treatment effect sizes (points for $\widehat{\tau }_{aipw}$ and $\widehat{\tau }_{tmle}$ are jittered due to overlapping values). The dashed line corresponds to the significance level of 0.05 to demonstrate Type-1 error control under the null hypothesis. (**B**) Power across different external control sample sizes (with RCT sample sizes and treatment effect size held fixed). While the SMA application has an external control sample size ∼50, other studies might have access to larger external control groups.

**Power and Type-1 error**: To highlight applicability to clinical trials, we simulate power under varying degrees of treatment effect sizes and external control sample sizes (Figure 2). We replicate Setting 5, modeling the (unknown) nuisance functions with Random Forests. Results are only depicted for $\widehat{\tau }_{aipw}$ and $\widehat{\tau }_{tmle}$ since constructing theoretically valid confidence intervals in this setting is unclear for $\widehat{\tau }_{om}$ and $\widehat{\tau }_{ipdw}$. When $\tau = 0$, both external control methods achieve satisfactory Type-1 error control ($\le \alpha = 5\%$). Across treatment effect sizes, power gains over $\widehat{\tau }_{rct}$ ranged from 13 to 65%. Furthermore, while our SMA application only has a pool of 50 external controls, more power gains are possible with larger sample sizes.

## 7 SMA EXAMPLE

**FIGURE 3:**
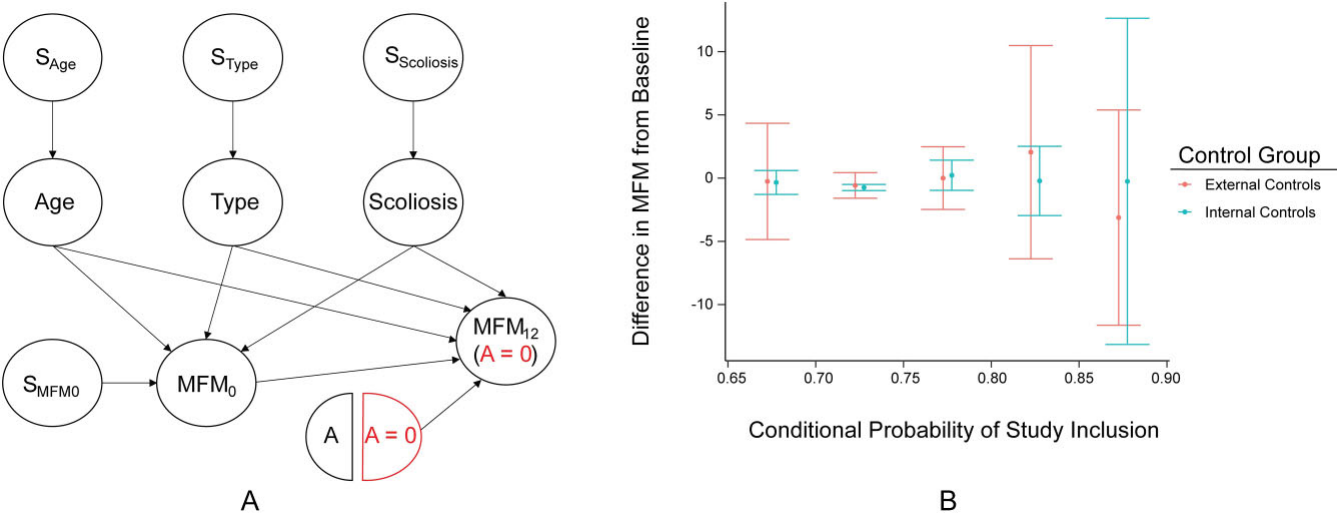
(**A**) Selection Diagram for the SMA hybrid trial. MFM$_0$ and MFM$_{12}$ correspond to the MFM scores at baseline and 12 months, respectively. Type refers to the SMA Type and A is the treatment (risdiplam). (**B**) Diagnostic of Assumption 4. Buckets were created based on $\widehat{\pi }_d(x)$ in increments of 0.05. Error bars correspond to 95% confidence intervals.

We demonstrate an implementation of the outlined causal inference framework using the SUNFISH (NCT02908685) trial. Further details of the trial are provided in the Supporting Information (Section 14). After restricting to complete cases with Type 2 or non-ambulant Type 3 SMA between the ages of 2-25, the RCT and external control samples contain 159 and 48 observations, respectively. The target parameter of interest is defined as $\tau = E[\Delta _{MFM}^1 | D = 1] - E[\Delta _{MFM}^0 | D = 1]$. We are interested in testing $H_0: \tau = 0$ versus $H_1: \tau \ne 0$. Based on discussions with the study team, we hypothesize the causal model depicted in Figure 3. The model is conservative in the sense that no assumptions are made about differences in distribution of the covariates between the RCT and the external control populations. Based upon this selection diagram, $E[\Delta _{MFM}^0 | D, X] = E[\Delta _{MFM}^0 | X]$, where $X = ($Age, SMA Type, Scoliosis, and MFM$_0)$. To assess covariate imbalance, we estimate $\widehat{\Delta }_{\pi _d} = 0.02$. This result, consistent with minimal covariate imbalance, increases confidence the historical placebo arm is sufficiently (pre-outcome) comparable to the internal controls.

We estimate $\tau$ with just the RCT data ($\widehat{\tau }_{rct}$) using AIPW and compare results to the four proposed estimators: $\widehat{\tau }_{om}$, $\widehat{\tau }_{ipdw}$, $\widehat{\tau }_{aipw}$, and $\widehat{\tau }_{tmle}$. For the doubly robust methods, the nuisance functions are fit using Random Forests (with cross-fitting using 10 folds) while linear models are used for $\widehat{\tau }_{om}$ and $\widehat{\tau }_{ipdw}$. Confidence intervals are based upon the nonparametric bootstrap for $\widehat{\tau }_{om}$ and $\widehat{\tau }_{ipdw}$ and from the closed-form formula for $\widehat{\tau }_{aipw}$ and $\widehat{\tau }_{tmle}$. All five methods provide similar estimates for $\tau$ and reject $H_0$ (Table 2). Overall, the confidence intervals for the four methods incorporating external controls have widths between 80% and 93% the width of the confidence interval using just the RCT data.

**TABLE 2:** Estimates of the causal effect for the SMA application.

Method	$\widehat{\tau }$	95% CI	*P*-value
$\widehat{\tau }_{rct}$	2.33	(0.82, 3.85)	0.002
$\widehat{\tau }_{om}$	1.93	(0.71, 3.13)	$< $ 0.001
$\widehat{\tau }_{ipdw}$	1.79	(0.54, 3.00)	0.002
$\widehat{\tau }_{aipw}$	1.92	(0.66, 3.18)	0.003
$\widehat{\tau }_{tmle}$	1.80	(0.54, 3.06)	0.005

To assess **A4**, we plot (Figure 3) the means of $\Delta _{MFM}$ for controls grouped by *D* and binning by $\widehat{\pi }_d(x)$. Under **A4**, we expect that $E[\Delta _{MFM} | D = 1, A = 0, \pi _d(x)] = E[\Delta _{MFM} | D = 0, A = 0, \pi _d(x)]$. We also estimated the asymptotic bias (via a plug-in estimate, Section 5.2) to be −0.19, which is small relative to the treatment effect and has a sign in the conservative direction. Altogether, the empirical evidence provides support that violations of **A4** for this external control arm are not responsible for the estimated benefit of risdiplman.

## 8 DISCUSSION

While it is well understood that deviations in characteristics and protocols between the RCT and external controls pose significant challenges to synthesizing information to estimate causal effects, the absence of a causal inference framework has hindered the clear communication of relevant concepts. The (causal) target parameter is rarely defined (Zhou and Ji, 2021 and Li et al., 2023 are notable exceptions) and discussions of bias center on practical heuristics (Pocock, 1976; Viele et al., 2014). Our proposed causal framework gives rise to an easy-to-interpret target parameter, encourages the embedding of assumptions and investigator knowledge into a causal graph, and allows bias to be explicitly defined as a property of the causal model or violation of the assumptions.

A limitation is the dependency upon **A4** to identify the causal effect, which can only be assessed after data collection and for which there might be little power to test empirically due to the small sample sizes commonplace in this setting. Furthermore, a null hypothesis of no difference might be of less interest than developing bounds based on the severity of the violations. While the methods discussed are applicable to continuous and binary outcomes, the extension to survival data would be an important contribution.

## Supplementary Material

ujae095_Supplemental_Files Web Appendices and code referenced in Sections 4-7 are available with this paper at the Biometrics website on Oxford Academic.

## Data Availability

The data that support the findings in this paper cannot be shared publicly due to the privacy of individuals that participated in the study.
