# Supplementary material for: A causal inference framework for leveraging external controls in hybrid trials
Source: Biometrics. 2024 Nov 8;80(4):ujae095. doi: 10.1093/biomtc/ujae095 (PMC11546536; doi:10.1093/biomtc/ujae095)
Supplement: ujae095_Supplemental_Files [file ujae095_supplemental_files.zip › Biometrics_Hybrid_Trial_External_Controls_Submission_Supp.pdf]

Supplementary Materials for "A Causal Inference Framework for Leveraging External Controls in Hybrid Trials" by Michael Valancius, Herbert Pang, Jiawen Zhu, Stephen R Cole, Michele Jonsson Funk, and Michael R Kosorok.

1. Relationship to Transportability/Generalizability

We expand on our discussion (Introduction of the main paper) about the relationship between the setting of our paper (hybrid trials with external controls) to transportability/generalizability. Unlike the causal inference task of transportability, the goal of a hybrid trial with external controls is not to transport inference about a causal effect from a study population to a target one. Instead, the task is somewhat reversed: external data are used for inference about the study population. Consistent with this goal, the target parameter that has been the primary focus of interest is the conventional target parameter in a traditional RCT. Because of these differing tasks, the statistical objectives differ. In hybrid trials, external data are used to increase efficiency of the study population's treatment effect estimate. Conversely, in transportability, external data are used to re-weight conditional average treatment effects. Despite these orthogonal targets of inference, the causal assumptions are similar in that they both require similarity between the potential outcome distributions in the two populations.

Letting S denote a binary indicator for selection into the study, the typical assumption made for transporting (or generalizing) results from a study to a target population is $E[Y^a|X, S = 1] = E[Y^a|X, S = 0]$ for $a \in \{0, 1\}$ (Dahabreh et al., 2019; Degtiar & Rose, 2023). Comparing this to **A4** and equating S with D, we observe that these assumptions are identical for $a = 0$, but no restrictions are placed on Y^1 since the external control cohort is never administered treatment $A = 1$.

2. Alternative Identifiability Criteria

In Section 3 of the main paper, we provided an example of assumptions that could be used to identify the causal effect of interest. The identification criteria listed, while sufficient for identifying τ, are not exhaustive and alternative strategies might be explored depending on the context. The assumption that $E[Y^0|D=1, X=x] = E[Y^0|D=0, X=x]$ for all x where $p(x|D=1) > 0$ might be overly strong. More generally, it may be the case that $E[Y^0|D=1, X] = E[Y^0|D=0, X] + u(X)$, where $u(X)$ captures the effects of unmeasured prognostic factors. One simplifying assumption might be that $E[Y^0|D=1, X] = E[Y^0|D=0, X] + \mu$ for some constant μ. This might be the case if there are baseline differences between internal and external controls not accounted for but independent of X. It is an open question of what efficiency gains are possible under this assumption.

3. Selection SWIG Examples

We expand on our discussion of the causal assumptions and proposed Selection SWIGs, highlighting their applicability through several examples of biases that might be present in this setting. We note that the proposed identifiability criteria make no assumptions about the distribution of (Y^1, D). Intuitively, no information from the external controls is informative about the outcome under the experimental treatment since no external controls receive the treatment.

When the causal assumptions are violated, τ cannot be computed even with infinite data, a bias sometimes referred to as systematic bias (Hernan & Robins, 2020). While sources of systematic bias in this setting, as well as heuristics to guard against it, have been previously proposed, the lack of a causal centering has led to criteria that can be vague or not directly target the underlying reason for the bias (Pocock, 1976). We encourage the viewpoint that discussions of bias should focus on the extent to which the outlined causal assumptions are

violated. Toward this goal, we highlight how common concerns with using external controls can threaten sound causal inference.

Geographic or temporal discrepancies between external controls patients and the study population can result in a cause of the outcome being imbalanced (hereafter meaning differing in distribution between the study population and the external control population). In the selection SWIGs, this is demarcated by the inclusion of a selection variable. It follows that the imbalance only threatens causal inference when the variable cannot be adjusted for, such as when it is unmeasured. Another concern, especially when the external control sample comes from a less quality-controlled source such as an EHR system, is measurement error. Because the external controls are all assigned the control, any form of mis-measurement will be differential with respect to the treatment.

[Figure 1 about here.]

4. The Role of Effect Modification

When constructing the causal graph $\mathbf{G}$, all variables with a causal effect on the potential outcome Y^0 should be included. Implicit in this construction is that the set of variables depends not only on the outcome Y but also on the choice of control treatment $A = 0$. A variable X is titled an effect modifier on the additive scale if $E[Y^1|X = x] - E[Y^0|X = x]$ is not constant over x, implying an interaction between the treatment and X.

When transporting the causal effect from one population to another, all effect modifiers must be adjusted for (Dahabreh et al., 2019; Cole & Stuart, 2010). In a hybrid trial design with external controls, the form of the control $a = 0$ dictates which variables have an effect on Y^0. Investigators might believe that a placebo control necessitates less adjustment (in the form of X) than an active control.

To aid in clarity, consider the following hypothetical example. An investigator can choose

among two control interventions: a placebo and an active control. The investigator is considering whether adjustments need to be made for two covariates that differ in distribution between the internal and external controls: age and sex. Sex is not believed to be have an effect on the outcome when patients are administered a placebo, but sex modifies the relationship between age and the outcome when the treatment is the active control. Figure 2 depicts this setting. If the placebo is used, only age is necessary for adjustment. If the active control is used, both age and sex are required. Ultimately, control interventions that are subject to greater degrees of effect modification pose heightened threats to the validity of the causal assumptions.

[Figure 2 about here.]

5. Identification of $E[Y^0|D = 1, X]$

In the following we prove the result in Section 3 that, under Assumptions 1-4, $E[Y^0|D = 1, X] = E[Y|A = 0, X]$:

$$\begin{aligned}
E[Y^0|D = 1, X] =& E[Y^0|D = 1, X]\big(\Pr(D = 1|A = 0, X) + \Pr(D = 0|A = 0, X)\big) \\
=& E[Y^0|D = 1, X]\Pr(D = 1|A = 0, X) \\
&+ E[Y^0|D = 0, X]\Pr(D = 0|A = 0, X) \\
=& E[Y^0|D = 1, A = 0, X]\Pr(D = 1|A = 0, X) \\
&+ E[Y^0|D = 0, A = 0, X]\Pr(D = 0|A = 0, X) \\
=& E[Y|D = 1, A = 0, X]\Pr(D = 1|A = 0, X) \\
&+ E[Y|D = 0, A = 0, X]\Pr(D = 0|A = 0, X) \\
=& E[Y|A = 0, X]. \quad \square
\end{aligned}$$

The second equality holds by Assumption 4, the third equality holds by Assumption 2 (and because $D = 0$ implies $A = 0$), and the fourth equality holds by Assumption 1.

6. Identification of $E[Y^0|D = 1, X]$ via the propensity formulation

The identification of the previous Section expresses $E[Y^0|D = 1]$ as the expectation over a population regression, which provides a natural motivation for the outcome modeling estimator. In the following, we also show that

$$E_{X|D=1}[E[Y^0|X, D = 1]] = \frac{1}{\Pr(D=1)} E\left[\frac{(1-A)}{(1-\pi_a(X))\pi_d(X)+(1-\pi_d(X))}\pi_d(X)Y\right],$$

which provides a motivation for the inverse propensity weighting estimator.

$$\begin{aligned}
E_{X|D=1}[E[Y^0|X, D = 1]] &= \int E[Y|A = 0, x]p(x|d = 1)dx \\
&= \frac{1}{\Pr(D = 1)} \int E[Y|A = 0, x]\pi_d(x)p(x)dx \\
&= \frac{1}{\Pr(D = 1)} \int\int y \frac{p(y|D = 1, A = 0, x)(1 - \pi_a(x))\pi_d(x)}{(1 - \pi_a(x))\pi_d(x) + (1 - \pi_d(x))} \pi_d(x)p(x)dydx \\
&+ \frac{1}{\Pr(D = 1)} \int\int y \frac{p(y|D = 0, A = 0, x)(1 - \pi_d(x))}{(1 - \pi_a(x))\pi_d(x) + (1 - \pi_d(x))} \pi_d(x)p(x)dydx \\
&= \frac{1}{\Pr(D = 1)} E\left[\frac{(1 - A)D + (1 - A)(1 - D)}{(1 - \pi_a(X))\pi_d(X) + (1 - \pi_d(X))} \pi_d(X)Y\right] \\
&= \frac{1}{\Pr(D = 1)} E\left[\frac{(1 - A)}{(1 - \pi_a(X))\pi_d(X) + (1 - \pi_d(X))} \pi_d(X)Y\right].
\end{aligned}$$

7. Discussion of Selection SWIGS and Proof of Theorem 1

The Selection SWIGs of Section 3 in the main manuscript are related to Bareinboim's selection diagrams (Bareinboim & Pearl, 2013), where we elect to use SWIGs (Richardson & Robins, 2013), as opposed to traditional causal diagrams, to facilitate reading off the conditional independence of Y^0 and S in the graph. While the Selection SWIGs draw

inspiration from Bareinboim's selection models, the latter are not readily applicable to this setting and their construction slightly differs (due to their assumption of independent errors of all covariates).

Proof of Theorem 1

To see the first statement, we observe that there must be no S_y present in the graph so that $p(y^0|z, d=1) = p(y^0|z, d=0)$. Let X^c denote $Z \backslash X$. Then we have the following, where in the third equality we make use of the observation from the previous line and the second step in the construction of $\mathbf{D}$:

$$\begin{aligned} p(y^0|x, d=1) &= \int p(y^0, x^c|x, d=1) dx^c \\ &= \int p(y^0|x, x^c, d=1) p(x^c|x, d=1) dx^c \\ &= \int p(y^0|x, x^c, d=0) p(x^c|x, d=0) dx^c \\ &= p(y^0|x, d=0) \qquad \square \end{aligned}$$

To see the second statement, let S_{Z_k} be a selection variable such that there is path from S_{Z_k} to Y^0 in $\mathbf{D}$. Divide the support of Z_k into two distinct regions, A_1 and A_0 so that, with probability 1, Z_k takes on values in A_1 if $D = 1$ and values in A_0 if $D = 0$. Furthermore, let the distribution of Y^0 only depend on Z_k and divide the support of Y into two distinct regions B_0 and B_1 such that Y always has larger values in B_0 than in B_1. Finally, let the distribution of Y^0 be such that, with probability 1, Y^0 takes on values in B_0 if $Z_k \in A_1$ and values in B_1 if $Z_k \in A_0$. Clearly $E[Y^0|X, D=1] > E[Y^0|X, D=0]$ for all $X \subset Z$. $\square$

8. Inverse-Propensity Weighting for Estimating the Average Treatment Effect on the Treated

In the following, we provide additional details on the Inverse-Propensity Weighting estimator of Section 4.2.

There is a direct connection between the proposed inverse propensity weighting method and the typical IPW method for estimating the ATT. The former is the empirical estimator of the following:

$$E\left[\frac{\Pr(D=1|X)}{\Pr(D=1)}\frac{(1-A)}{\Pr(A=0|X)}Y\right].$$

The latter, when estimating $E[Y^0|A=1]$, is the empirical estimator of the following:

$$E\left[\frac{1}{\Pr(A=1)}\frac{\Pr(A=1|X)(1-A)}{\Pr(A=0|X)}Y\right].$$

We observe that the only difference is the ratios $\frac{\Pr(A=1|X)}{\Pr(A=1)}$ and $\frac{\Pr(D=1|X)}{\Pr(D=1)}$. However, these ratios are equivalent as long as $\Pr(A=1|X,D=1)=\Pr(A=1|D=1)$, as can be seen in the following:

$$\begin{aligned}\frac{\Pr(A=1|X)}{\Pr(A=1)} &= \frac{\Pr(A=1|X,D=1)\Pr(D=1|X)+\Pr(A=1|X,D=0)\Pr(D=0|X)}{\Pr(A=1|D=1)\Pr(D=1)+\Pr(A=1|D=0)\Pr(D=0)}\\ &= \frac{\Pr(A=1|X,D=1)\Pr(D=1|X)+0}{\Pr(A=1|D=1)\Pr(D=1)+0}\\ &= \frac{\Pr(D=1|X)}{\Pr(D=1)}.\end{aligned}$$

Therefore, we see that the inverse propensity score estimator estimates the same quantity as the inverse propensity method for calculating the ATT. The distinguishing characteristic, based on the factorization of $\Pr(A=1|X)$ into $\pi_a(X)\pi_d(X)$, is whether one estimates $\Pr(A=1|X)$ or $\pi_d(X)$.

8.1 *Handling Extreme Weights in* $\widehat{\mu}_{0,ipdw}$

When the sample size is small, the weights used in $\widehat{\mu}_{0,ipdw}$ can be extreme, leading to high variance and point estimates that might fall outside the support of Y. One remedy is to construct a bounded estimator that normalizes the weights:

$$\widetilde{\mu}_{0,ipdw} = \left(\frac{1}{n} \sum_{i=1}^{n} \widehat{W}_i^{ipdw} \right)^{-1} \sum_{i=1}^{n} \widehat{W}_i^{ipdw} Y_i.$$

8.2 *Matching Based on* $\widehat{\pi}_d$

An alternative approach to balance the treated and control participants is matching. A common strategy when X is high dimensional, as is likely in this setting, is to match treated and control units based upon the propensity score (Rosenbaum & Rubin, 1983). The propensity score $\pi(x) := \Pr(A = 1|X = x)$ is a balancing score: $A \perp X|\pi(X)$ and therefore $Y^a \perp A|\pi(X)$ if $Y^a \perp A|X$ (Imbens & Rubin, 2015). A natural generalization to this setting is matching RCT individuals with external controls based upon the study propensity score $\pi_d(x)$. Analogous to the treatment propensity score, the study propensity score is a balancing score in the sense that $D \perp X|\pi_d(X)$, as shown in the following Section. When combining $D \perp X|\pi_d(X)$ with Assumption 4, we have mean-exchangeability given $\pi_d(X)$. That is, $E[Y^0|\pi_d(X), D] = E[Y^0|\pi_d(X)]$.

Because $E[Y^0|\pi_d(x)]$ is the same for internal and external controls for all x with support in the study population, matching external controls to study participants with the same values of $\pi_d(X)$ and then taking the difference in outcomes between the treated and controls produces an unbiased estimate of the target parameter. However, in practice, $\pi_d(x)$ must be estimated and there will be no exact matches on $\widehat{\pi}_d(X)$, leading to bias that depends on the accuracy of the estimator and the amount of discrepancy in treatment propensity scores of matched individuals (Abadie & Imbens, 2016).

8.3 *Balancing Score Property*

To show that $D \perp X|\pi_d(X)$, we will equivalently show that $\Pr(D = 1|X, \pi_d(X)) = \Pr(D = 1|\pi_d(X))$.

The left hand side of the equation is $\pi_d(X)$, since

$$\Pr(D=1|X,\pi_d(X)) = \Pr(D=1|X) = \pi_d(X).$$

Furthermore, by conditioning on X, we can see that the right hand side is also equal $\pi_d(X)$:

$$\begin{aligned}\Pr(D=1|\pi_d(X)) &= E[D|\pi_d(X)] \\ &= E[E[D|\pi_d(X),X]|\pi_d(X)] \\ &= E[\pi_d(X)|\pi_d(X)] \\ &= \pi_d(X).\end{aligned}$$

8.4 *Mean-exchangeability given $\pi_d(x)$*

Mean-exchangeability given $\pi_d(x)$ provides a basis for which we can assess **A4** through a one-dimensional summary. To see that mean-exchangeability holds given $\pi_d(x)$, we will now show that $E[Y^0|\pi_d(X),D] = E[Y^0|\pi_d(X)]$. This is shown in the following:

$$\begin{aligned}E[Y^0|\pi_d(X),D] &= \int E[Y^0|x,\pi_d(x),d]p(x|\pi_d(x),d)dx \\ &= \int E[Y^0|x,\pi_d(x)]p(x|\pi_d(x),d)dx \\ &= \int E[Y^0|x,\pi_d(x)]p(x|\pi_d(x))dx \\ &= E[Y^0|\pi_d(X)] \qquad \square\end{aligned}$$

The second-to-last equality uses the fact that $p(x|\pi_d(x),d) = p(x|\pi_d(x))$ because $\Pr(D = 1|X=x,\pi_d(x)) = \Pr(D=1|\pi_d(x))$.

9. TMLE Implementation

Additional details on the TMLE procedure described in Section 4.3 of the main paper are provided in the following. The target parameter τ is the contrast between $E[Y^1|D=1]$ and

$E[Y^0|D=0]$. As before, we can identify $E[Y^1|D=1]$ with $E_{X|D=1}[E[Y|A=1,X]]$ and $E[Y^0|D=1]$ with $E_{X|D=1}[E[Y|A=0,X]]$. Therefore, a simple plug-in estimator of τ is given by:

$$\widehat{\tau}_{plugin} = \frac{1}{n_1}\sum_{i=1}^{n} D_i\big(\widehat{m}_1(X_i) - \widehat{m}_0(X_i)\big)$$

A TMLE estimator of τ is also a plug-in estimator, but instead of using an original estimator $\widehat{m}_a(x)$ of $E[Y|A=a,X=x]$, it updates $m_a(x)$ to produce $m_a^*(x)$, which is then plugged into the above empirical average. A comprehensive discussion of TMLE can be found in (Van der Laan & Rose, 2011). In the following discussion, we demonstrate its application to this particular problem. First, we will demonstrate a parametric fluctuation that can be used to update our estimate of $m_a(x)$ using a particular loss function that is valid for the conditional mean. We then show that the derivative of our fluctuation loss, evaluated when the fluctuation is zero, equals the efficient influence curve. Therefore, the TMLE estimator solves the efficient influence curve estimating equation.

Define $\widetilde{Y} \equiv (Y-a)/(b-a)$, where $(a,b) = (\min(Y), \max(Y))$. Then, we can define $\widetilde{\tau}$ as $E_{X|D=1}[E[\widetilde{Y}|X,A=1]] - E_{X|D=1}[E[\widetilde{Y}|X,A=0]]$. It follows that $\widetilde{Y} \in [0,1]$ and $\tau = \widetilde{\tau}(b-a)$ so that inference for $\widetilde{\tau}$ can be mapped to inference for τ. Initial estimators $\widehat{m}_0(x)$ and $\widehat{m}_1(x)$ are constructed for $\widetilde{m}_0(x) \equiv E[\widetilde{Y}|X=x,A=0]$ and $\widetilde{m}_1(x) \equiv E[\widetilde{Y}|X=x,A=1]$. While these estimators traditionally have been constructed with SuperLearner (Van der Laan, 2007), this need not be the case, and our simulations model these with Random Forests for a simpler, consistent comparison of the methods. Furthermore, let $\widehat{\pi}_d(x)$, $\widehat{\pi}_a(x)$, and $\widehat{r}(x)$ be estimators of $\pi_d(x)$, $\pi_a(x)$, and $r(x)$.

To update $\widehat{m}_a(x)$, we follow (Gruber & van der Laan, 2010) and consider a parametric fluctuation that guarantees the model respects the range of the outcome. More specifically,

observe that $m_a(x)$ can be expressed as $(1+\exp\{-\log(m_a(x)/(1-m_a(x)))\})^{-1}$. We define the following fluctuation model

$$m_a(x)(\epsilon) \equiv \frac{1}{1+\exp\{-\log(m_a(x)/(1-m_a(x)))+\epsilon h(d,a,x)\}},$$

where the clever covariate $h(D,A,X)$ is defined as

$$h(D,A,X) \equiv \frac{1}{q}\left\{\frac{DA}{\widehat{\pi}_a(X)} - \frac{D(1-A)\widehat{\pi}_d(X)+(1-D)(1-A)\widehat{\pi}_d(X)\widehat{r}(X)}{\widehat{\pi}_d(X)(1-\widehat{\pi}_a(X))+(1-\widehat{\pi}_d(X))\widehat{r}(X)}\right\}.$$

The motivation for this fluctuation model is to ensure that the fluctuations always respect the range of the outcome. Next, we consider the loss function $L(\epsilon) \equiv L_1(\epsilon_1) + L_2(\epsilon_2)$, where

$$L^1(\epsilon_1) \equiv Y\log(m_1(X)) + (1-Y)m_0\log(1-m_0(X))$$

and

$$L^2(\epsilon_2) \equiv -\log\big\{1+\epsilon_2\frac{D}{q}(m_1(X)-m_0(X)-\tau)\big\}P_X.$$

Under this loss function, it can be shown that $\frac{d}{d\epsilon}L(\epsilon)|_{\epsilon=0} = \phi(O)$. Iteratively minimizing this loss with respect to ϵ produces the TMLE estimator. However, since the update is taken on the logit scale, i.e. $\text{logit}(m_a(x))(\epsilon) = \text{logit}(m_a(x)) + \epsilon h(d,a,x)$ and by definition of a maximum likelihood estimator, the MLE of ϵ after one fluctuation of $\widehat{m}_a(x)$ is 0, so that convergence occurs after only one step and therefore only one iteration of updates is needed.

To conduct the above procedure, after fitting initial models for $m_a(x), \pi_d(x)$, and $\pi_a(x)$, we fit a logistic regression model of $\widetilde{Y}$ on $h(D,A,X)$ with offset given by the logit of $\widehat{m}_A(X)$, ignoring that $\widetilde{Y}$ is not binary. For each observation O_i, we can get predictions from this logistic regression model under the setting that $A=1$ and the setting that $A=0$. This defines our updated models $\widehat{m}_1^*(x)$ and $\widehat{m}_0^*(x)$. Then, our estimator for τ is given by:

$$\widehat{\tau}_{tmle} = \left(\frac{1}{b-a}\right)\frac{1}{n_1}\sum_{i=1}^{n} D_i\big(\widehat{m}_1^*(X_i) - \widehat{m}_0^*(X_i)\big).$$

10. Semiparametric Theory

In order to provide a proof of Theorem 2, we first provide an introduction to some essential elements of semiparametric theory. The ideas in the following are based upon a growing literature on the property of causal inference estimators that leverage machine learning methods to non-parametrically estimate nuisance functions (Kennedy, 2022).

More formally, we can think of τ as a map from $\mathcal{P}$ to $\mathbb{R}$, where $\mathcal{P}$ is a semiparametric model containing the true distribution $\mathbb{P}$ and whose only restriction is that $E[Y^0|X, D = 1] = E[Y^0|X, D = 0]$ almost surely.[1] As before, we can express $\tau(P)$ as the following:

$$\tau(P) = E_{P_{X|D=1}}[E_P[Y|A = 1, X]] - E_{P_{X|D=1}}[E_P[Y|A = 0, X]].$$

We note that this expression depends upon P only through $E_P[Y|A = 0, X]$, $P_{X|D=1}$, and $E_P[Y|A = 1, X]$. In this semiparametric model, the efficient influence curve for the parameter $\tau(P)$ is given by (Li et al., 2023) to be

$$\phi(O;\mathbb{P}) = \frac{1}{q}\left\{D(m_1(X)-m_0(X)-\tau)+\frac{DA}{\pi_a(X)}(Y-m_1(X))-W(A,D,X)(Y-m_0(X))\right\}, \quad (1)$$

where $q \equiv \int \pi_d(x)d\mathbb{P}$, $r(X) \equiv \text{Var}(Y^0|X, D = 1)/\text{Var}(Y^0|X, D = 1)$ and where

$$W(A,D,X) \equiv \frac{D(1-A)\pi_d(X) + (1-D)\pi_d(X)r(X)}{\pi_d(X)(1-\pi_a(X)) + (1-\pi_d(X))r(X)}.$$

To analyze the doubly-robust estimators when using machine learning models for the nuisance functions, we make use of the Von-Mises expansion (Van der Vaart, 2000; Kennedy, 2022; Hines et al., 2022):

[1] While $\pi_a(x)$ is also known, it is ancillary to the estimation of τ in the sense that the efficiency bound for τ is the same regardless of whether of not $\pi_a(x)$ is known.

$$\tau(\overline{P}) - \tau(P) = \int \phi(o, \overline{P}) d(\overline{P} - P)(o) + R_2(\overline{P}, P).$$

where $P\phi(O, P) = 0$, $P\phi(O, P)^2 < \infty$, and $R_2(\overline{P}, P)$ is a second order remainder term. We wish to consider the performance of an estimator that substitutes $\widehat{\mathbb{P}}$ for $\mathbb{P}$, where $\widehat{\mathbb{P}}$ denotes a model in which we have used estimators for $E_P[Y|A - 0, X]$, $P_{X|D=1}$, and $E_P[Y|A = 1, X]$. We first consider the decomposition of the plug-in estimator that plugs in $\widehat{\mathbb{P}}$ for $\mathbb{P}$. Based upon the von-Mises expansion, we have that it can be expressed in the following way:

$$\tau(\widehat{\mathbb{P}}) - \tau(\mathbb{P}) = -\mathbb{P}\phi(O, \widehat{\mathbb{P}}) + R_2(\widehat{\mathbb{P}}, \mathbb{P})$$

The term $-\mathbb{P}\phi(O, \widehat{\mathbb{P}})$ is a first-order bias term, and a common way of removing this bias is through adding an estimate of it: $\mathbb{P}_n\phi(O, \widehat{\mathbb{P}})$. This new estimator, $\tau(\widehat{\mathbb{P}}) + \mathbb{P}_n\phi(O, \widehat{\mathbb{P}})$, is exactly equal to $\widehat{\tau}_{aipw}$:

$$\begin{aligned}
\tau(\widehat{\mathbb{P}}) + \mathbb{P}_n\phi(O, \widehat{\mathbb{P}}) &= \big(\sum_{i=1}^{n} D_i\big)^{-1} \sum_{i=1}^{n} D_i\big(\widehat{m}_1(X_i) - \widehat{m}_0(X_i)\big) + \sum_{i=1}^{n} \phi(O_i, \widehat{\mathbb{P}}) \\
&= \big(\sum_{i=1}^{n} D_i\big)^{-1} \sum_{i=1}^{n} D_i\big(\widehat{m}_1(X_i) - \widehat{m}_0(X_i)\big) - \frac{1}{q}\sum_{i=1}^{n} D_i\big(\widehat{m}_1(X_i) - \widehat{m}_0(X_i)\big) \\
&+ \frac{1}{n}\frac{1}{\widehat{q}}\sum_{i=1}^{n}\Big\{D_i\big(\widehat{m}_1(X_i) - \widehat{m}_0(X_i)\big) + \frac{D_iA_i}{\widehat{\pi}_a(X_i)}(Y_i - \widehat{m}_1(X_i)) \\
&- \widehat{W}(A_i, D_i, X_i)(Y_i - \widehat{m}_0(X_i))\Big\} \\
&= \frac{1}{n}\frac{1}{\widehat{q}}\sum_{i=1}^{n}\Big\{D_i\big(\widehat{m}_1(X_i) - \widehat{m}_0(X_i)\big) + \frac{D_iA_i}{\widehat{\pi}_a(X_i)}(Y_i - \widehat{m}_1(X_i)) \\
&- \widehat{W}(A_i, D_i, X_i)(Y_i - \widehat{m}_0(X_i))\Big\} \\
&= \widehat{\tau}_{aipw}
\end{aligned}$$

To observe the asymptotic equivalence between $\widehat{\tau}_{aipw}$ and $\widehat{\tau}_{tmle}$, we note that $\widehat{\tau}_{aipw}$ is defined

by solving the estimating equation $\mathbb{P}_n\{\phi(O,\widehat{\mathbb{P}},\tau)\}=0$. Conversely, $\widehat{\tau}_{tmle}$ is defined as $\tau(\widehat{\mathbb{P}}^*)=\tau(\widehat{\mathbb{P}}^*)+\mathbb{P}_n\{\phi(O,\widehat{\mathbb{P}}^*)\}$ since $\widehat{\mathbb{P}}^*$ is defined so that $\mathbb{P}_n\{\phi(O,\widehat{\mathbb{P}}^*)\}=0$.

We consider the following decomposition based on the von-Mises expansion (Kennedy, 2022):

$$\widehat{\tau}_{dr}-\tau(\mathbb{P})=\tau(\widehat{\mathbb{P}})+\mathbb{P}_n\phi(O,\widehat{\mathbb{P}})-\tau(\mathbb{P}) \tag{2}$$

$$=\big(\mathbb{P}_n-\mathbb{P}\big)\{\phi(O,\widehat{\mathbb{P}})\}+R_2(\widehat{\mathbb{P}},\mathbb{P}) \tag{3}$$

$$=\big(\mathbb{P}_n-\mathbb{P}\big)\{\phi(O,\mathbb{P})\}+(\mathbb{P}_n-\mathbb{P})\{\phi(O,\widehat{\mathbb{P}})-\phi(O,\mathbb{P})\}+R_2(\widehat{\mathbb{P}},\mathbb{P}). \tag{4}$$

In the above, $\widehat{\tau}_{dr}$ stands for either $\widehat{\tau}_{aipw}$ or $\widehat{\tau}_{tmle}$, where it is understood that $\widehat{\mathbb{P}}$ is $\widehat{\mathbb{P}}^*$ when referring to $\widehat{\tau}_{tmle}$.

By the central limit theorem, the first term converges to a normally distributed random variable with mean 0 and variance equal to the efficiency bound (scaled by $1\backslash n$). Hence, if the other terms are asymptotically negligible compared to the first term ($o_{\mathbb{P}}(1\backslash\sqrt{n})$, then $\widehat{\tau}_{dr}$ is $\sqrt{n}-$consistent, asymptotically normal, and semiparametric efficient.

We first show how to control the second term (often termed the empirical process term). While Donsker conditions can be used to show this term is asymptotically negligible, we opt for cross-fitting (sometimes called sample-splitting) due to its simplicity and weaker conditions. In sample splitting, the full data, $O^N=(O_1,...,O_K)$ is randomly split into K (approximately) even sized folds. For each fold k of size N_k, the nuisance functions of the influence curve ($m_0(x),\pi_d(x)$, etc.) are estimated using only observations not contained in the kth fold, producing an estimator $\widehat{\mathbb{P}}_{-k}$ of $\mathbb{P}$. Altogether, this produces K estimators: $(\widehat{\mathbb{P}}_{-1},...,\widehat{\mathbb{P}}_{-K})$. Then $\widehat{\tau}_{dr}$ is constructed by first fitting K estimators $\widehat{\tau}^k_{dr}$ where, for the kth estimator, the influence curve takes $\widehat{\mathbb{P}}_{-k}$ instead of $\widehat{\mathbb{P}}$ as its argument and is averaged over the data in the kth fold. Then, $\widehat{\tau}_{dr}$ is the weighted average of these K estimators $\widehat{\tau}^k_{dr}$, with the weights corresponding to the proportion of the total observations in the fold. More details

can be found in Kennedy (2022). The following lemma provides the necessary tool to use cross-fitting to control the second term.

Lemma 1 (Kennedy, 2022). Let $\widehat{f}(o)$ be a function estimated from a sample $O^N = (O_{n+1}, ..., O_N)$, and let $\mathbb{P}_n$ denote the empirical measure over $(O_1, ..., O_n)$, which is independent of O^N. Then:

$$(\mathbb{P}_n - \mathbb{P})(\widehat{f} - f) = O_{\mathbb{P}}\left(\frac{||\widehat{f}_n - f||}{\sqrt{n}}\right)$$

Therefore, to show that $(\mathbb{P}_n - \mathbb{P})\{\phi(O, \widehat{\mathbb{P}}) - \phi(O, \mathbb{P})\}$ is asymptotically negligible, it suffices to show that $\phi(O, \widehat{\mathbb{P}}_{-k})$ converges to $\phi(O, \mathbb{P})$ in $L_2(\mathbb{P})$ norm for each k:

$$||\phi(\widehat{\mathbb{P}}_{-k}) - \phi(\mathbb{P})|| \equiv \left(\int \big(\phi(o, \widehat{\mathbb{P}}_{-k}) - \phi(o, \mathbb{P})\big)^2 d\mathbb{P}(o)\right)^{1/2} = o_{\mathbb{P}}(1).$$

Below, we present conditions for which this is true. For readability, we omit the distinction between $\widehat{\mathbb{P}}_{-k}$ and $\widehat{\mathbb{P}}$.

Lemma 2 Suppose that:

- $||\widehat{m}_0(x) - m_0(x)||$, $||\widehat{m}_1(x) - m_1(x)||$, $||\widehat{\pi}_a(x) - \pi_a(x)||$, $||\widehat{\pi}_d(x) - \pi_d(x)||$, and $||\widehat{r}(x) - r(x)||$ are all $o_{\mathbb{P}}(1)$.
- $\widehat{\pi}_d(x)$ and $\pi_d(x)$ are greater than some $\epsilon_d > 0$ with probability 1
- $\widehat{\pi}_a(x)$ and $\pi_a(x)$ are greater than some $\epsilon_a > 0$ and less than $1 - \epsilon_a < 1$ with probability 1
- $\beta(x) \equiv \pi_d(x)\big(1 - \pi_a(x)\big) + \big(1 - \pi_d(x)\big)r(x)$ and $\widehat{\beta}(x) \equiv \widehat{\pi}_d(x)\big(1 - \widehat{\pi}_a(x)\big) + \big(1 - \widehat{\pi}_d(x)\big)\widehat{r}(x)$ are greater than some $\delta > 0$ with probability 1.
- $0 < r(X) < R$ with probability 1.
- $A(Y - m_1(X)) \leqslant M_1$ and $(1 - A)(Y - m_0(X)) \leqslant M_0$ with probability 1.

Then $||\phi(\widehat{\mathbb{P}}) - \phi(\mathbb{P})||^2 = o_{\mathbb{P}}(1)$.

Proof

$$\phi(\widehat{\mathbb{P}}) - \phi(\mathbb{P}) = \frac{1}{q}\big\{D\big(\widehat{m}_1(x) - m_1(x)\big) - D\big(\widehat{m}_0(x) - m_0(x)\big) + \frac{DA}{\widehat{\pi}_a(x)}\big(Y - \widehat{m}_1(x)\big)$$
$$- \frac{DA}{\pi_a(x)}\big(Y - m_1(x)\big) - \widehat{W}(A, D, X)\big(Y - \widehat{m}_0(x)\big) + W(A, D, X)\big(Y - m_0(x)\big)\big\}$$
$$\equiv \frac{1}{q}\big(A - B\big).$$

Evaluating these terms separately, we obtain that:

$$\frac{1}{q}A = \frac{1}{q}D\big(\widehat{m}_1(x) - m_1(x) - \frac{A}{\widehat{\pi}_a(x)}\widehat{m}_1(x) + \frac{A}{\pi_a(x)}m_1(x) + \frac{A}{\widehat{\pi}_a(x)}Y - \frac{A}{\pi_a(x)}Y\big)$$
$$= \frac{1}{q}D\big\{\big(\widehat{m}_1(x) - m_1(x)\big)\big(1 - \frac{A}{\pi_a(x)}\big) + \frac{A}{\widehat{\pi}_a(x)\pi_a(x)}\big(Y - \widehat{m}_1(x)\big)\big(\pi_a(x) - \widehat{\pi}_a(x)\big)\big\}.$$

Similarly, we find that:

$$\frac{1}{q}B = \frac{1}{q}\big\{D\widehat{m}_0(x) - Dm_0(x) + \widehat{W}(A, D, X)\big(Y - \widehat{m}_0(x)\big) - W(A, D, X)\big(Y - m_0(x)\big)\big\}$$
$$= \frac{1}{q}\big\{\big(\widehat{m}_0(x) - m_0(x)\big)\big(D - W(A, D, X)\big) + \widehat{W}(A, D, X)\big(Y - \widehat{m}_0(x)\big)$$
$$- W(A, D, X)\big(Y - \widehat{m}_0(x)\big)\big\}$$
$$= \frac{1}{q}\big\{\big(\widehat{m}_0(x) - m_0(x)\big)\big(D - W(A, D, X)\big) + \big(Y - \widehat{m}_0(x)\big)\big(\widehat{W}(A, D, X) - W(A, D, X)\big)\big\}.$$

By the sub-additive property of norms, we then have that:

$$
\begin{aligned}
||\phi(\widehat{\mathbb{P}}) - \phi(\mathbb{P})|| \leqslant \frac{1}{q}\Big\{ & ||D\big(\widehat{m}_1(x) - m_1(x)\big)\big(1 - \frac{A}{\pi_a(x)}\big)|| + ||\frac{A}{\widehat{\pi}_a(x)\pi_a(x)}\big(Y - \widehat{m}_1(x)\big)\big(\pi_a(x) - \widehat{\pi}_a(x)\big)|| \\
& + ||\big(\widehat{m}_0(x) - m_0(x)\big)\big(D - W(A,D,X)\big)|| \\
& + ||\big(Y - \widehat{m}_0(x)\big)\big(\widehat{W}(A,D,X) - W(A,D,X)\big)||\Big\} \\
& \leqslant \frac{1}{q}\Big\{\frac{1}{\epsilon_a}||\widehat{m}_1(x) - m_1(x)|| + ||\frac{M_1}{\epsilon_a^2}\big(\pi_a(x) - \widehat{\pi}_a(x)\big)|| + \frac{R+1}{\epsilon_a}||\widehat{m}_0(x) - m_0(x)|| \\
& + ||M_0\big[D\big(\frac{\widehat{\pi}_d(x)}{\widehat{\beta}(x)} - \frac{\pi_d(x)}{\beta(x)}\big) + (1-D)\big(\frac{\widehat{\pi}_d(x)\widehat{r}(x)}{\widehat{\beta}(x)} - \frac{\pi_d(x)r(x)}{\beta(x)}\big)\big]||\Big\} \\
& = o_{\mathbb{P}}(1) + \frac{1}{q}||M_0\Big\{D\big(\frac{\widehat{\pi}_d(x)}{\widehat{\beta}(x)} - \frac{\pi_d(x)}{\beta(x)}\big) + (1-D)\big(\frac{\widehat{\pi}_d(x)\widehat{r}(x)}{\widehat{\beta}(x)} - \frac{\pi_d(x)r(x)}{\beta(x)}\big)\Big\}||.
\end{aligned}
$$

Therefore, it only remains to show that the second term, which comes from the simplification of $\widehat{W}(A,D,X) - W(A,D,X)$, is $o_{\mathbb{P}}(1)$. We show this by evaluating the two parts of the term separately. The first part can be decomposed into terms that are all $o_{\mathbb{P}}(1)$ in the following way:

$$
\begin{aligned}
\frac{\widehat{\pi}_d(x)}{\widehat{\beta}(x)} - \frac{\pi_d(x)}{\beta(x)} &= \frac{\widehat{\pi}_d(x)\beta(x) - \pi_d(x)\widehat{\beta}(x)}{\beta(x)\widehat{\beta}(x)} \\
&= \frac{\pi_d(x)\widehat{\pi}_d(x)\big(\widehat{\pi}_a(x) - \pi_a(x)\big) + \big(\widehat{\pi}_d(x) - \pi_d(x)\big) + \pi_d(x)\widehat{\pi}_d(x)\big(\widehat{r}(x) - r(x)\big)}{\beta(x)\widehat{\beta}(x)}.
\end{aligned}
$$

The second part has a similar decomposition:

$$
\begin{aligned}
\frac{\widehat{\pi}_d(x)\widehat{r}(x)}{\widehat{\beta}(x)} - \frac{\pi_d(x)r(x)}{\beta(x)} &= \frac{\widehat{\pi}_d(x)\widehat{r}(x)\beta(x) - \pi_d(x)r(x)\widehat{\beta}(x)}{\beta(x)\widehat{\beta}(x)} \\
&= \frac{\pi_d(x)\widehat{\pi}_d(x)\big(\widehat{r}(x) - r(x)\big) + \pi_d(x)\widehat{\pi}_d(x)\big(\widehat{\pi}_a(x)r(x) - \pi_a(x)\widehat{r}(x)\big)}{\beta(x)\widehat{\beta}(x)} \\
&+ \frac{r(x)\widehat{r}(x)\big(\widehat{\pi}_d(x) - \pi_d(x)\big)}{\beta(x)\widehat{\beta}(x)}.
\end{aligned}
$$

Combining these results, we obtain that:

$$
\begin{aligned}
&||M_0\Big\{D\big(\frac{\widehat{\pi}_d(x)}{\widehat{\beta}(x)}-\frac{\pi_d(x)}{\beta(x)}\big)+(1-D)\big(\frac{\widehat{\pi}_d(x)\widehat{r}(x)}{\widehat{\beta}(x)}-\frac{\pi_d(x)r(x)}{\beta(x)}\big)\Big\}||\\
&\leqslant \frac{1}{\delta^2}\Big\{||\widehat{\pi}_a(x)-\pi_a(x)||+||\widehat{\pi}_d(x)-\pi_d(x)||+||\widehat{r}(x)-r(x)||\\
&||\widehat{r}(x)-r(x)||+||\widehat{\pi}_a(x)r(x)-\pi_a(x)\widehat{r}(x)||+R^2||\widehat{\pi}_d(x)-\pi_d(x)||\Big\}\\
&=o_{\mathbb{P}}(1)+\frac{1}{\delta^2}\Big\{||r(x)\big(\widehat{\pi}_a(x)-\pi_a(x)\big)||+||\pi_a(x)\big(r(x)-\widehat{r}(x)\big)||\Big\}\\
&=o_{\mathbb{P}}(1)\quad\square
\end{aligned}
$$

Corollary 1: Let the assumptions given by Lemma 2 hold for $\widehat{\mathbb{P}}_{-k}$ for each k. Then the cross-fit doubly robust estimator $\widehat{\tau}_{dr}$ has the property that:

$$\widehat{\tau}_{dr}-\tau(\mathbb{P})=(\mathbb{P}_n-\mathbb{P})\{\phi(O,\mathbb{P})\}+R_2^*(\widehat{\mathbb{P}},\mathbb{P})+o_{\mathbb{P}}(1/\sqrt{n}) \tag{5}$$

where $R_2^*(\widehat{\mathbb{P}},\mathbb{P})=\sum_{k=1}^{K}\frac{N_k}{n}R_2(\widehat{\mathbb{P}}_{-k},\mathbb{P})$

We now turn our attention to the third term, $R_2^*(\widehat{\mathbb{P}},\mathbb{P})$, for which the following lemma provides a closed-form expression of.

Lemma 3: Let $\widehat{\mathbb{P}}$ be defined by estimators $\widehat{\pi}_a(x)$, $\widehat{\pi}_d(x)$, $\widehat{m}_1(x)$, $\widehat{m}_0(x)$, and $\widehat{r}(x)$. Furthermore, let $\mathbb{P}D=\widehat{\mathbb{P}}D=\frac{n_1}{n}$. Then we have the following:

$$
\begin{aligned}
R_2(\widehat{\mathbb{P}},\mathbb{P})=\int\Big\{&(m_0(x)-m_1(x))+(\widehat{m}_1(x)-\widehat{m}_0(x))+\frac{\pi_a(x)}{\widehat{\pi}_a(x)}(m_1(x)-\widehat{m}_1(x))\\
&-\frac{\widehat{\pi}_d(x)}{\pi_d(x)}(m_0(x)-\widehat{m}_0(x))\frac{\pi_d(x)(1-\pi_a(x))+(1-\pi_d(x))\widehat{r}(x)}{\widehat{\pi}_d(x)(1-\widehat{\pi}_d(x))+(1-\widehat{\pi}_d(x))\widehat{r}(x)}\Big\}f(x|d=1)dx
\end{aligned}
$$

Proof:

By the Von-Mises expansion and the form of $\phi(O,P)$, we have that:

$$R_2(\widehat{\mathbb{P}},\mathbb{P})=\tau(\widehat{\mathbb{P}})-\tau(\mathbb{P})+\int\phi(o,\widehat{\mathbb{P}})d(\mathbb{P}-\widehat{\mathbb{P}})(o)$$

Since $\int\phi(o,\widehat{\mathbb{P}})d\widehat{\mathbb{P}}(o)=0$, this gives that

$$R_2(\widehat{\mathbb{P}}, \mathbb{P}) = \tau(\widehat{\mathbb{P}}) - \tau(\mathbb{P}) + \int \phi(o, \widehat{\mathbb{P}}) d\mathbb{P}(o)$$

The rest follows from expanding the specific expressions:

$$\begin{aligned}
R_2(\widehat{\mathbb{P}}, \mathbb{P}) &= \tau(\widehat{\mathbb{P}}) - \tau(\mathbb{P}) + \int \phi(o, \widehat{\mathbb{P}}) d\mathbb{P}(o) \\
&= -\tau(\mathbb{P}) + \frac{1}{\widehat{\mathbb{P}}D} \int \left\{ d(\widehat{m}_1(x) - m_1(x)) + \frac{da}{\widehat{\pi}_a(x)}(y - \widehat{m}_1(x)) - \widehat{w}(a,d,x)(y - \widehat{m}_0(x)) \right\} d\mathbb{P} \\
&= -\tau(\mathbb{P}) + \int (\widehat{m}_1(x) - m_1(x)) f(x|d=1)dx + \int \frac{\pi_a(x)}{\widehat{\pi}_a(x)}(\widehat{m}_1(x) - m_1(x)) f(x|d=1)dx \\
&- \int \frac{\widehat{\pi}_d(x)}{\pi_d(x)}(m_0(x) - \widehat{m}_0(x)) \frac{\pi_d(x)(1-\pi_a(x)) + (1-\pi_d(x))\widehat{r}(x)}{\widehat{\pi}_d(x)(1-\widehat{\pi}_d(x)) + (1-\widehat{\pi}_d(x))\widehat{r}(x)} f(x|d=1)dx \\
&= \int \left\{ (m_0(x) - m_1(x)) + (\widehat{m}_1(x) - \widehat{m}_0(x)) + \frac{\pi_a(x)}{\widehat{\pi}_a(x)}(m_1(x) - \widehat{m}_1(x)) \right. \\
&= \left. - \frac{\widehat{\pi}_d(x)}{\pi_d(x)}(m_0(x) - \widehat{m}_0(x)) \frac{\pi_d(x)(1-\pi_a(x)) + (1-\pi_d(x))\widehat{r}(x)}{\widehat{\pi}_d(x)(1-\widehat{\pi}_d(x)) + (1-\widehat{\pi}_d(x))\widehat{r}(x)} \right\} f(x|d=1)dx \quad \square
\end{aligned}$$

Now that we have a closed-form expression for $R_2(\widehat{\mathbb{P}}, \mathbb{P})$, we establish conditions under which it is asymptotically negligible.

Lemma 4: Assume that $||m_1(x) - \widehat{m}_1(x)|| ||\pi_a(x) - \widehat{\pi}_a(x)|| = o_{\mathbb{P}}(a_n)$, $||\widehat{m}_0(x) - m_0(x)|| ||\pi_a(x) - \widehat{\pi}_a(x)|| = o_{\mathbb{P}}(b_n)$, and $||\widehat{m}_0(x) - m_0(x)|| ||\pi_d(x) - \widehat{\pi}_d(x)|| = o_{\mathbb{P}}(c_n)$, where the norm is defined with respect to the study distribution. Furthermore, assume that $0 < \epsilon \leqslant \widehat{\pi}_a(x)$ and $\widehat{r}(x) \leqslant M < \infty$ for all x such that $f(x|d=1) > 0$. Then $R_2(\widehat{\mathbb{P}}, \mathbb{P}) = o_{\mathbb{P}}(\max(a_n, b_n, c_n))$

Proof

By Lemma 3, we have that:

$$\begin{aligned}
R_2(\widehat{\mathbb{P}}, \mathbb{P}) = \int \Bigg\{ & (m_0(x) - m_1(x)) + (\widehat{m}_1(x) - \widehat{m}_0(x)) + \frac{\pi_a(x)}{\widehat{\pi}_a(x)}(m_1(x) - \widehat{m}_1(x)) \\
& - \frac{\widehat{\pi}_d(x)}{\pi_d(x)}(m_0(x) - \widehat{m}_0(x)) \frac{\pi_d(x)(1-\pi_a(x)) + (1-\pi_d(x))\widehat{r}(x)}{\widehat{\pi}_d(x)(1-\widehat{\pi}_d(x)) + (1-\widehat{\pi}_d(x))\widehat{r}(x)} \Bigg\} f(x|d=1)dx.
\end{aligned}$$

We first consider the expressions involving $m_1(x)$ and $\widehat{m}_1(x)$.

$$
\begin{aligned}
&\int \Big\{(\widehat{m}_1(x) - m_1(x)) + \frac{\pi_a(x)}{\widehat{\pi}_a(x)}(m_1(x) - \widehat{m}_1(x))\Big\} f(x|d=1)dx \\
&= \int \big(\widehat{m}_1(x) - m_1(x)\big)\big(\pi_a(x) - \widehat{\pi}_a(x)\big)\frac{1}{\widehat{\pi}_a(x)} f(x|d=1)dx \\
&\leqslant \int \big(\widehat{m}_1(x) - m_1(x)\big)\big(\pi_a(x) - \widehat{\pi}_a(x)\big)\frac{1}{\epsilon} f(x|d=1)dx \\
&\leqslant ||m_1(x) - \widehat{m}_1(x)||||\pi_a(x) - \widehat{\pi}_a(x)||\frac{1}{\epsilon} \\
&= o_{\mathbb{P}}(a_n).
\end{aligned}
$$

The second to last inequality follows from Cauchy-Schwartz. Next, we consider the remainder expressions of $R_2(\widehat{\mathbb{P}}, \mathbb{P})$. Letting $\lambda(x) = \widehat{\pi}_d(x)\pi_d(x)(1 - \pi_a(x)) + (1 - \pi_d(x))\widehat{r}(x)$ and $\widehat{\lambda}(x) = \pi_d(x)\widehat{\pi}_d(x)(1 - \widehat{\pi}_d(x)) + (1 - \widehat{\pi}_d(x))\widehat{r}(x)$, we obtain the following:

$$
\begin{aligned}
&\int \Big\{\big(m_0(x) - \widehat{m}_0(x)\big) - \frac{\widehat{\pi}_d(x)}{\pi_d(x)}(m_0(x) - \widehat{m}_0(x))\frac{\pi_d(x)(1 - \pi_a(x)) + (1 - \pi_d(x))\widehat{r}(x)}{\widehat{\pi}_d(x)(1 - \widehat{\pi}_d(x)) + (1 - \widehat{\pi}_d(x))\widehat{r}(x)}\Big\} f(x|d=1)dx \\
&= \int \big(m_0(x) - \widehat{m}_0(x)\big)\big(1 - \frac{\lambda(x)}{\widehat{\lambda}(x)}\big) f(x|d=1)dx \\
&= \int \big(m_0(x) - \widehat{m}_0(x)\big)\big(\widehat{\lambda}(x) - \lambda(x)\big)\frac{1}{\widehat{\lambda}(x)} f(x|d=1)dx \\
&\leqslant \int \big(m_0(x) - \widehat{m}_0(x)\big)\big(\widehat{\lambda}(x) - \lambda(x)\big)\frac{1}{M} f(x|d=1)dx \\
&\leqslant ||\widehat{m}_0(x) - m_0(x)||\widehat{\lambda}(x) - \lambda(x)||\frac{1}{M} \\
&= ||\widehat{m}_0(x) - m_0(x)||||\widehat{\pi}_d(x)\pi_d(x)\big(\pi_a(x) - \widehat{\pi}_a(x)\big) + \big(\pi_d(x) - \widehat{\pi}_d(x)\big)\widehat{r}(x)||\frac{1}{M} \\
&\leqslant ||\widehat{m}_0(x) - m_0(x)||\pi_a(x) - \widehat{\pi}_a(x)|||| + ||\widehat{m}_0(x) - m_0(x)||||\pi_d(x) - \widehat{\pi}_d(x)||\frac{1}{M} \\
&= o_{\mathbb{P}}(b_n) + o_{\mathbb{P}}(c_n). \quad \square
\end{aligned}
$$

We now provide a full statement of Theorem 2 from the main paper, whose proof immediately follows from the direct application of Lemmas 2 and 4 and the following regularity conditions.

Theorem 2 Suppose that the regularity conditions in Lemma 2 hold for $\widehat{\mathbb{P}}_{-k}$ for each

k. Furthermore, assume that $||m_1(x) - \widehat{m}_1(x)||||\pi_a(x) - \widehat{\pi}_a(x)|| = o_{\mathbb{P}}(1\backslash\sqrt{n})$, $||\widehat{m}_0(x) - m_0(x)||||\pi_a(x) - \widehat{\pi}_a(x)|| = o_{\mathbb{P}}(1\backslash\sqrt{n})$, and $||\widehat{m}_0(x) - m_0(x)||||\pi_d(x) - \widehat{\pi}_d(x)|| = o_{\mathbb{P}}(1\backslash\sqrt{n})$, where the norm is defined with respect to the study distribution and this holds for each cross-fit model. Furthermore, assume that $0 < \epsilon \leqslant \widehat{\pi}_a(x)$ and $\widehat{r}(x) \leqslant M < \infty$ for all x such that $f(x|d = 1) > 0$. Then:

$$\sqrt{n}(\widehat{\tau}_{dr} - \tau) \xrightarrow{d} N(0, E[\phi(O, \mathbb{P})]).$$

11. Upper Bound on Asymptotic Bias

As mentioned in Section 5 of the main paper, upper bounds can be derived for the asymptotic bias that might be useful in settings where the noise is small compared to the treatment effect. The asymptotic bias, when the estimators for the nuisance functions are consistent and $r(x) = 1$, is $E[\Pr(D = 0|A = 0, X)u(X)|D = 1]$, which is upper-bounded (when $u(X)$ and $Pr(D = 0|A = 0, X)$ are independent) by $\Pr(D = 0|A = 0)E[u(X)|D = 1]$. We will establish an upper bound on $E[u(X)|D = 1]$.

Let $\sigma^2 = E[(Y - E[Y|A = 0, X])^2|A = 0, D = 1]$ and $\sigma_d^2 = E[(Y - E[Y|A = 0, D = 1, X])^2|A = 0, D = 1]$. Because $\sigma^2 \geqslant \sigma_d^2$ and $\sigma_d^2 > 0$, $\sigma^2 - \sigma_d^2 \leqslant \sigma^2$. Furthermore, $\sigma^2 - \sigma_d^2 = E[(E[Y|A = 0, D = 1, X] - E[Y|A = 0, X])^2|A = 0, D = 1]$. Using Jensen's inequality, this implies that

$$E[(E[Y|A = 0, D = 1, X] - E[Y|A = 0, X])|A = 0, D = 1] \leqslant \sigma.$$

Furthermore, we have that, when $\pi_d(X)$ is independent of $u(X)$, that

$$\begin{aligned} &E[(E[Y|A = 0, D = 1, X] - E[Y|A = 0, X])|A = 0, D = 1] \\ &= E[(1 - \pi_d(X))|D = 1] \times E[u(X)|A = 0, D = 1]. \end{aligned}$$

Combining these results, we get that

$$E[u(X)|D=1] \leqslant E[(1-\pi_d(X))|D=1]^{-1}\sigma.$$

Both of the quantities on the right hand side can be evaluated empirically, and the bound may have implications when it is reasonably small compared to the treatment effect. The interpretation is that, if adjustments for just X explain the majority of variability in Y (given $A=0$), then there is not much unaccounted for residual variability that would be consistent with large values of $u(X)$.

12. Simulation Details

We provide further details on the simulation setup. In the simulations, three covariates, X_1, X_2, X_3, were generated independently. X_1 followed a Bernoulli(0.5) distribution while X_2 and X_3 both followed a Uniform(-1,1) distribution. In our simulation, we aimed to understand the properties of the estimators when $(X_1, X_2, X_3) \not\perp D$. Therefore, conditional upon these covariates, the study indicator D was drawn from a Bernoulii distribution with mean $\pi_d(X_1, X_2, X_3)$, where

$$\pi_d(X_1, X_2, X_3) = \text{logit}^{-1}\Big(f_{1a}(X_1) + f_{1b}(1-X_1) + f_2(X_2) + f_3(X_3)\Big).$$

We chose the basis functions in the following way:

$$\begin{aligned} f_{1a}(x) &= \log\left(\tfrac{0.7}{0.3}\right)x \\ f_{2a}(x) &= \log\left(\tfrac{0.3}{0.7}\right)x \\ f_2(x) &= \log\left\{2\frac{\frac{3}{7}(1.5-x^2)}{\frac{1}{6}+x^2}\right\} \\ f_3(x) &= \log\left\{\frac{\frac{\sqrt{\pi}}{\text{erf}(1)}e^{-x^2}}{\frac{1.5-e^{-x^2}}{3-\sqrt{\pi}\text{erf}(1)}}\right\}. \end{aligned}$$

This formulation allows us to directly control the relative ratio of the densities in the RCT and external control populations ($\frac{p(x_1,x_2,x_3|d=1)}{p(x_1,x_2,x_3|d=0)}$), where the ratio of the marginal densities is given by the fractions inside the logarithms) and therefore characterize differences in the distributions between the two populations. Under this simulation, when $D = 1$, $X_1 = 1$ more commonly and both X_2 and X_3 have greater density around 0. Because all of the covariates are bounded, the uniform overlap assumption on π_d is satisfied.

For the study sample, one third of individuals were randomly assigned $A = 0$ (control) and two thirds were assigned $A = 1$ (treatment), independent of (X_1, X_2, X_3). If $D = 0$, then $A = 0$. The outcome Y was generated as follows:

$$Y = 10 + X_1 - 2.5X_2^2 - 2.5X_3^2 + A\big(0.7 + 0.5X_1 + 0.2(1 - X_2^2)\big) + \epsilon.$$

Additionally, $\epsilon \sim N(0, 1)$ and is independent of any of the other variables.

To estimate the nuisance functions, two approaches were considered. When investigators had access to transformed versions of the variables (so that the logits and expected outcome were linear functions of the variables), a logistic regression model with main effects was used for $\pi_d(x)$ and a linear regression model with main effects was used for $m_0(x)$ (and fit just to samples with $A = 0$). In these scenarios, the parameter estimates were consistent for their true values. When investigators did not have access to the transformed variables (Settings 2, 3, and 5), Random Forests were used via the randomForest package in R. For both nuisance models, forests with 500 trees and a *mtry* parameter (number of variables randomly sampled as candidates at each split) of 2 were fit. The maximum number of nodes for the tree was selected as 5 for the outcome model and 10 for the study propensity model. While the number of trees was chosen based on computational considerations, the maximum number of nodes and *mtry* parameter were chosen based on cross-validation from the first simulation iteration. We found that these hyperparameters tended to be fairly consistent in

this simulation setting across different data set generations. All simulations were performed using R 4.1.0.

Confidence intervals for $\widehat{\tau}_{om}$ and $\widehat{\tau}_{ps}$ were constructed using the nonparametric bootstrap (percentile method). For $\widehat{\tau}_{aipw}$ and $\widehat{\tau}_{tmle}$, the estimators asymptotically follow a normal distribution with variance equal to the variance of the efficient influence function. Empirical estimates of this variance were calculated using the fitted nuisance functions and estimates of the average treatment effect.

13. Simulation Results: Confidence Interval Coverage

We discuss the simulation results related to confidence interval coverage. As seen in Table 1, all methods have approximately correct coverage when the models are correctly specified, with the two doubly-robust methods having tighter intervals. When one model was misspecified and the other was fit with a data-adaptive model, the two doubly-robust estimators had mixed results, exhibiting sufficient coverage in one setting but not the other. This is likely due to the fact that bias of the estimator and the validity of the confidence intervals depends on the product of the rates of convergence for the two nuisance models. If one nuisance function is completely misspecified (Settings 2 and 3), the other must converge at $\sqrt{n}$-rate. However, if both estimators converge slowly (Setting 5), nominal coverage is still attained. Surprisingly, the bootstrap confidence intervals for $\widehat{\tau}_{om}$ achieve the targeted coverage in Setting 5. However, there is a lack of a theoretical basis for this result, and other simulations have demonstrated poor confidence interval coverage when using machine learning with singly-robust models (Naimi, Mishler, & Kennedy, 2023).

[Table 1 about here.]

14. SUNFISH Trial

In this Section, we provide additional details on the real-world motivating example. SUNFISH (NCT02908685) (Mercuri et al., 2022) was a two-part multi-site randomized placebo-controlled trial designed to investigate the efficacy of risdiplam. In Part 2 of the study, 180 patients with Type 2 and non-ambulant Type 3 SMA were randomized 2:1 to receive risdiplam or placebo for 12 months. At the completion of 12 months, patients originally randomized to placebo were switched to risdiplam. While the SUNFISH trial did not incorporate external controls into its analysis, we use it as a hypothetical example of a trial that, were it conducted in the future, could benefit from the incorporation of external controls. The external controls considered in this paper are from the placebo arm of a Phase 2 trial of olesoxime (NCT01302600). The trial is temporally and geographically similar, which could help justify the conditional mean exchangeability assumption. After restricting to complete cases with Type 2 or non-ambulant Type 3 SMA between the ages of 2-25, the RCT and external control samples contain 159 and 48 observations, respectively. While the original study performed a longitudinal analysis, for simplicity we restrict our attention to the change from baseline at 12 months. The primary endpoint of interest was the change in Motor Function Measure (MFM) at 12 months (Δ_{MFM}). Several different scales exist for MFM. In order to ensure the comparability of MFM scores between the placebo group of the external control and the patients in SUNFISH, the change in baseline in the MFM total score is derived from MFM32 for all patients aged 6 years or above and MFM20 for all patients aged less than 6 years.

References

Abadie, A., & Imbens, G. W. (2016). Matching on the estimated propensity score. Econometrica, 84(2), 781-807.

Bareinboim, E., & Pearl, J. (2013). A general algorithm for deciding transportability of experimental results. *Journal of Causal Inference*, 1(1), 107-134.

Cole, S. R., & Stuart, E. A. (2010). Generalizing evidence from randomized clinical trials to target populations: the ACTG 320 trial. *American Journal of Epidemiology*, 172(1), 107-115.

Dahabreh, I. J., Robins, J. M., Haneuse, S. J., & Hernán, M. A. (2019). Generalizing causal inferences from randomized trials: counterfactual and graphical identification. https://arxiv.org/abs/1906.10792

Degtiar, I., & Rose, S. (2023). A review of generalizability and transportability. *Annual Review of Statistics and Its Application*, 10, 501-524.

Gruber, S., & van der Laan, M. J. (2010). A targeted maximum likelihood estimator of a causal effect on a bounded continuous outcome. *The International Journal of Biostatistics*, 6(1).

Hernán MA, Robins JM (2020). *Causal Inference: What If.* Boca Raton: Chapman & Hall/CRC

Hines, O., Dukes, O., Diaz-Ordaz, K., & Vansteelandt, S. (2022). Demystifying statistical learning based on efficient influence functions. *The American Statistician*, 76(3), 292-304.

Imbens, G. W., & Rubin, D. B. (2015). *Causal inference in statistics, social, and biomedical sciences.* Cambridge University Press.

Kennedy, E. H. (2022). *Semiparametric doubly robust targeted double machine learning: a review.* https://arxiv.org/abs/2203.06469

Li, X., Miao, W., Lu, F., & Zhou, X. H. (2023). Improving efficiency of inference in clinical trials with external control data. *Biometrics*, 79(1), 394-403.

Mercuri, E., Deconinck, N., Mazzone, E. S., Nascimento, A., Oskoui, M., Saito, K., et al;. (2022). Safety and efficacy of once-daily risdiplam in type 2 and non-ambulant type

3 spinal muscular atrophy (SUNFISH part 2): a phase 3, double-blind, randomised, placebo-controlled trial. *The Lancet Neurology*, 21(1), 42-52.

Naimi, A. I., Mishler, A. E., & Kennedy, E. H. (2023). Challenges in obtaining valid causal effect estimates with machine learning algorithms. *American Journal of Epidemiology*, 192(9), 1536-1544.

Pocock, S. J. (1976). The combination of randomized and historical controls in clinical trials. *Journal of Chronic Diseases*, 29(3), 175-188.

Richardson, T. S., & Robins, J. M. (2013). Single world intervention graphs (SWIGs): A unification of the counterfactual and graphical approaches to causality. https://csss.uw.edu/research/working-papers/single-world-intervention-graphs-swigs-unification-counterfactual-and

Rosenbaum, P. R., & Rubin, D. B. (1983). The central role of the propensity score in observational studies for causal effects. *Biometrika*, 70(1), 41-55.

Van der Vaart, A.W. (2000) *Asymptotic Statistics*, volume 3. Cambridge University Press

Van der Laan, M. J., Polley, E. C., & Hubbard, A. E. (2007). Super learner. *Statistical Applications in Genetics and Molecular Biology*, 6(1).

Van der Laan, M. J., & Rose, S. (2011). *Targeted learning* (Vol. 1, No. 3). New York: Springer.

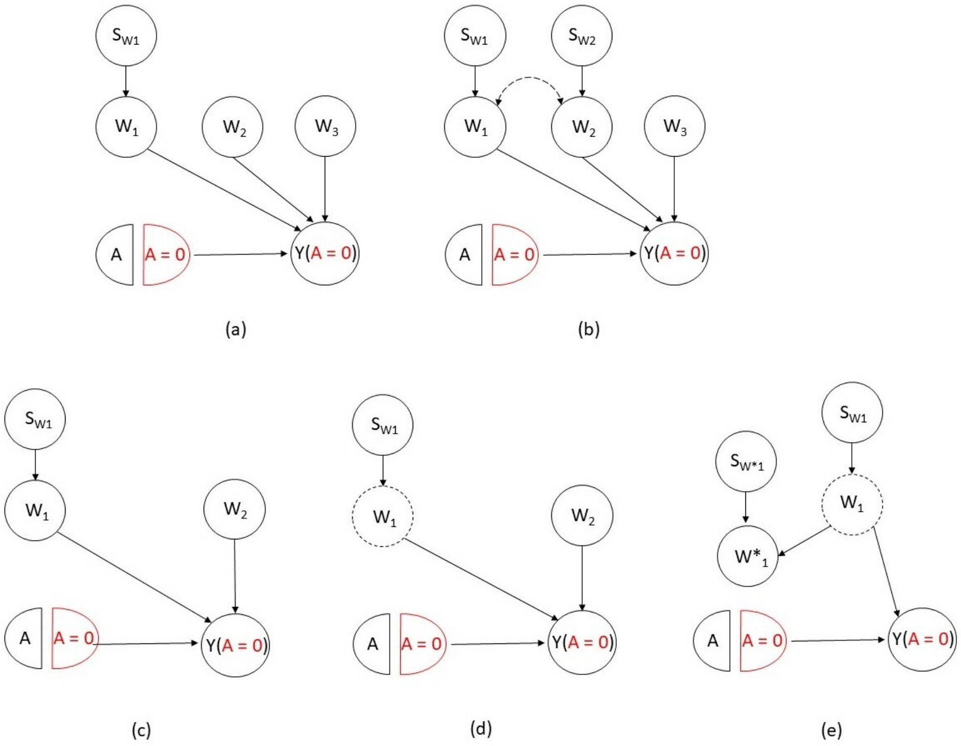

Figure 1. Examples of Selection SWIGs. (a) By Theorem 1, $Y^0 \perp D|W_1$. (b) By Theorem 1, $Y^0 \perp D|W_1, W_2$. (c) W_1 and W_2 are causes of Y^0, but only W_1 is imbalanced. Once W_1 is conditioned on, S is independent of Y^0, and therefore W_1 is sufficient for adjustment. (d) When W_1 is unmeasured (dotted lines), causal bias is incurred. (e) W_1 should be adjusted for, but W_1^* is the mis-measured variable available to investigators. The node $S_{W_1^*}$ pointing into W_1^* represents the belief that the quality of W_1^* (how well it measures W_1) differs between external controls and the study sample.

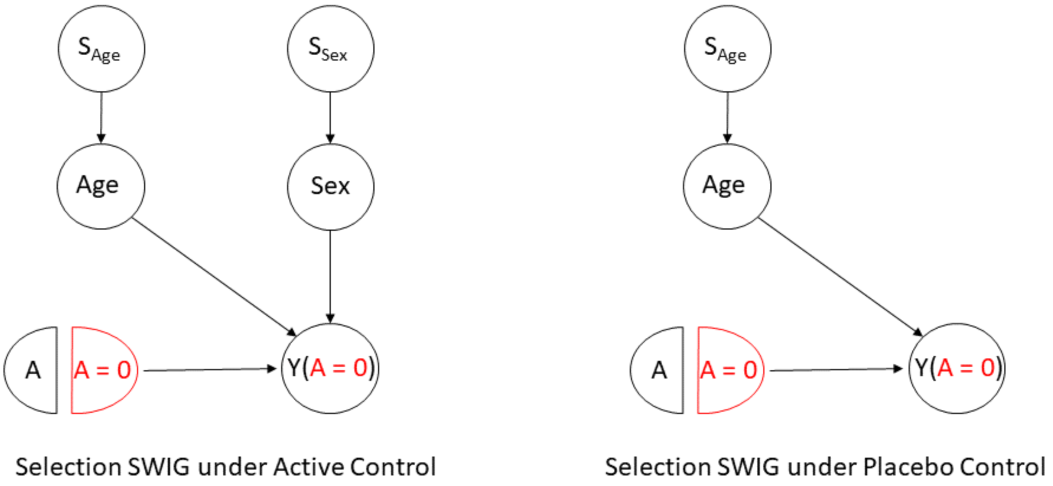

Figure 2. Example of the role of effect modification on the adjustment set.

Table 1

Results (bias, mean-squared error, and 95% confidence interval coverage) of the simulation. In Setting 1, all nuisance functions are estimated with correctly specified linear models. In Setting 2, an incorrectly specified linear model is used for $\pi_d(x)$ while Random Forests are used for $m_a(x)$. In Setting 3, incorrectly specified linear models are used for $m_a(x)$ while a Random Forest is used for $\pi_d(x)$. In Setting 4, incorrectly specified linear models are used for both $m_a(x)$ and $\pi_d(x)$. Both $m_a(x)$ and $\pi_d(x)$ are modeled with Random Forests in Setting 5

	$\widehat{\tau}_{rct}$			$\widehat{\tau}_{om}$			$\widehat{\tau}_{ipdw}$			$\widehat{\tau}_{aipw}$			$\widehat{\tau}_{tmle}$		
	Bias	MSE	Cov.	Bias	MSE	Cov.	Bias	MSE	Cov.	Bias	MSE	Cov.	Bias	MSE	Cov.
Setting 1	-1.8e-03	0.31	0.96	2.5e-04	**0.22**	0.93	-2.9e-03	0.24	0.93	-4.2e-04	0.23	0.95	**6.6e-06**	**0.22**	0.96
Setting 2	**3.4e-02**	0.31	0.95		—	—	4.0e-01	0.38	0.86	2.0e-01	**0.25**	0.94	2.2e-01	**0.25**	0.94
Setting 3	**3.6e-02**	0.34	0.95	4.1e-01	0.38	0.85	—	—	—	1.1e-01	**0.24**	0.96	6.8e-02	**0.24**	0.96
Setting 4	**2.5e-02**	**0.34**	0.96	4.0e-01	0.39	0.85	4.0e-01	0.39	0.86	4.2e-01	0.42	0.86	4.1e-01	0.40	0.88
Setting 5	**-4.7e-03**	0.32	0.95	2.2e-01	0.24	0.93	4.8e-02	0.22	0.94	5.8e-02	**0.21**	0.96	6.3e-02	**0.21**	0.96
